# Comorbidities associated with a clinically-recognized delirium diagnosis in the hospital using real world data

**DOI:** 10.1038/s43856-025-00986-5

**Published:** 2025-07-22

**Authors:** Lay Kodama, Sarah R. Woldemariam, Alice S. Tang, Yaqiao Li, John Kornak, Isabel Elaine Allen, Eva Raphael, Tomiko T. Oskotsky, Marina Sirota

**Affiliations:** 1 Bakar Computational Health Sciences Institute, University of California San Francisco, San Francisco, CA USA; 2 Medical Scientist Training Program, University of California San Francisco, San Francisco, CA USA; 3 Psychiatry Department, University of California San Diego, La Jolla, CA USA; 4 Department of Epidemiology and Biostatistics, University of California, San Francisco, San Francisco, CA USA; 5 Department of Family and Community Medicine, University of California San Francisco, San Francisco, CA USA; 6 Division of Clinical Informatics and Digital Transformation, Department of Medicine, University of California San Francisco, San Francisco, CA USA; 7 Department of Pediatrics, University of California San Francisco, San Francisco, CA USA

**Keywords:** Disorders of consciousness, Dementia

## Abstract

**Background:**

Delirium is a mental condition defined as fluctuating disturbances in attention, awareness, and cognition. It is often seen in older, hospitalized patients and is currently hard to predict, with long- and short-term outcomes being detrimental to patients.

**Methods:**

We leveraged electronic health records (EHR) to identify 7492 UCSF patients and 19,417 UC health system patients with an inpatient delirium diagnosis and the same number of control patients without delirium. We used the Fisher’s exact test with multiple corrections for the association studies and the Cox regression model for the longitudinal analyses.

**Results:**

Here we show significant associations between comorbidities or laboratory values and an inpatient delirium diagnosis, including metabolic abnormalities and psychiatric diagnoses. Some associations are sex-specific, including dementia subtypes and infections. We further explore the associations with anemia and bipolar disorder by conducting longitudinal analyses from the time of first diagnosis to development of delirium, demonstrating a significant relationship across time. Finally, we show that an inpatient delirium diagnosis leads to increased risk of mortality.

**Conclusions:**

These results demonstrate the powerful application of the EHR to shed insights into prior diagnoses and laboratory values that could help predict development of inpatient delirium and the importance of sex when making these assessments.

## Plain language summary

Delirium is a serious and harmful condition characterized by a change in mental function compared to baseline, with symptoms often including confusion, impaired attention, and perceptual disturbances. It is a condition often seen in older, hospitalized patients and is currently hard to predict. Here we use data from medical records of patients diagnosed with delirium to better understand what other diagnoses are associated with delirium and show that one episode of delirium in the hospital can increase a patient’s risk of death long-term. These findings portray the detrimental outcome of delirium and emphasize the need for more awareness and research about the condition to find better ways to predict and prevent this condition in the hospital.

## Abstract (web summary)

Kodama et al. use electronic health record data of patients with and without a delirium diagnosis to identify the comorbidities and laboratory values associated with inpatient delirium diagnoses. Longitudinal analysis also show that an inpatient delirium diagnosis is associated with an increased risk of mortality independent of comorbidity burden.

## Introduction

Delirium is a clinical diagnosis defined as fluctuating disturbances in attention, awareness, and cognition that develops over a short period of time^1^. It is highly prevalent among older inpatient populations, with an estimated incidence of 9%^2^ and prevalence of 23% in hospitalized older adults, though this estimate ranges widely, from 4% to 54%, depending on the clinical setting and patient population^3,4^. Both long- and short-term outcomes of delirium are detrimental to patients, distressing to caregivers, and a burden on the healthcare system^5–7^. Notably, patients who are diagnosed with delirium have an increased risk of long-term mortality, though this finding has not been consistent across different studies, potentially due to being underpowered^5,8–10^. It is unclear whether delirium is an independent risk factor for mortality or whether other unadjusted, confounding factors are being captured by a delirium diagnosis, such as overall baseline disease burden, and are influencing mortality risk^5^. Medications for treating delirium are largely for symptomatic management and have not been shown to have clinical benefits and in fact may lead to worse outcomes, especially for older patients^11^. Prevention is the most effective strategy and often involve non-pharmacological interventions, such as reorientation and cognitive stimulation^12^, making it imperative to predict which patients may develop delirium using predictive tools to focus prevention efforts for high-risk patients.

Though prevalent, delirium remains challenging to study and predict given its heterogenous clinical nature and diverse risk factors^3^. Current prediction tools have variable predictive capabilities, with one systematic review finding an area under the receiver operating curve range from 0.52 to 0.94^13^. These models have other limitations including validation in small sample sizes or data collected from one institution that may not be generalizable^14,15^. These models incorporate well-studied risk factors such as existing cognitive impairment and severity of chronic illnesses, but less apparent risk factors also need to be identified to improve these predictive tools^13,16,17^.

Importantly, sex is a major risk modifier for many neurological diseases, with some evidence of sex-differences in delirium^18–20^. For instance, one study found hypoactive delirium to be more common in female compared to male patients^19^. Though clinical sex differences have been well-documented in dementia and cognitive impairment—major risk factors for delirium—whether sex differences exist in delirium remains largely unstudied due to the lack of sex-stratified studies.

One strategy to overcome these limitations is to employ large-scale, comprehensive real-world data from electronic health records (EHR) combined with robust computational approaches. Data from the EHR have been used to better understand and phenotype complex diseases such as Alzheimer’s disease, type 2 diabetes, and preterm birth^21–23^. Such deep phenotyping studies can shed insights into clinical risk factors and subtypes of disease as well as point to potential new biological pathways that may be involved in disease.

In this study, we leverage EHRs from two databases across California to identify differential comorbidities and laboratory test results prior to a patient’s first inpatient admission for delirium. We find patients with delirium have enrichment of diagnoses related to diseases of the nervous system, mental health and behavioral disorders, and acute diagnoses such as metabolic diseases and infections with differential laboratory test results corroborating several of these diagnoses including anemia and depression. Importantly, in our sex-stratified analyses, we find female and male patients diagnosed with delirium have sex-specific associations with distinct infections and subtypes of dementia. We further analyze specific comorbidities in a longitudinal manner and show specific diagnoses including bipolar disorder and anemia increase the probability of developing an inpatient delirium diagnosis, complementing the association study and providing granularity into the time course from comorbidity diagnosis to delirium diagnosis. Lastly, we find one inpatient delirium diagnosis is associated with worse mortality outcomes, even after adjusting for important covariates such as comorbidity burden.

## Methods

### Experimental model and study participation details

Analyses of UCSF and University of California Data Discovery Platform (UCDDP) de-identified EMR data were performed by UCSF employees under Institutional Review Board (IRB) approvals from UCSF, UCD, UCI, UCLA, and UCSD. All clinical data were de-identified and so a requirement for written informed consent to be obtained from people whose data was included was waived by the institutions. Patient cohorts were identified using the UCSF de-identified and UC-wide HIPAA-compliant limited data set OMOP EHR databases. The UCSF dataset included over five million patients from January 1, 1982 to February 20, 2023 while the UC-wide dataset included over seven million patients from January 1, 2012 to April 19, 2023 from 4 sites (UC San Diego, UC Los Angeles, UC Irvine, and UC Davis). Patients with inpatient delirium were identified using the OMOP concept ID 373995, SNOMED code 2776000 (corresponding to “Delirium”), filtered for first-time diagnosis of delirium during an inpatient stay (i.e., ‘visit of interest’). Codes from the International Classification of Diseases (ICD, e.g., ICD-9 and ICD-10 codes) and other vocabularies (e.g., Logical Observation Identifiers Names and Codes (LOINC) and Current Procedural Terminology (CPT)) are mapped to Observational Medical Outcomes Partnership (OMOP) IDs within the OMOP Common Data Model (CDM) to ensure consistency across various coding systems. OMOP IDs standardize data for large-scale research, serve as a bridge between different coding systems like ICD, and enable more comprehensive analyses. Please see for more details: https://github.com/OHDSI/Vocabulary-v5.0/wiki/Standardized-Vocabularies. The inpatient control cohort with no delirium diagnosis was identified through propensity score (PS) matching (matchit R^24^) by a generalized linear model at a 1:1 ratio using a nearest-neighbor method and the following matching criteria: assigned sex, patient-reported race, estimated age at admission, years in EHR prior to visit, total number of comorbidities and inpatient visits prior to the visit of interest, stay length, stay type (ICU vs. non-ICU), death during admission, and UC location (for UC-wide dataset). Age exclusion criteria was not utilized. Propensity score matching was used similar to other previously published works using clinical datasets^21,25–27^. For sex stratification, we utilized reported sex and excluded nonbinary and other categories due to low sample size, similar to a prior study^21^. See Supplementary Data 1 for cohort demographic details.

### Differential comorbidity analysis

Comorbidities were identified prior to the visit of interest with the earliest entry of every diagnosis. All patients and their comorbidities were visualized using uniform manifold approximation and projection (UMAP) using the R-implementation of the umap-learn package from Python. UMAP is a form of dimensionality reduction to plot high-dimensional data into a lower-dimension using machine learning techniques^28^. In this case, a table was created with all distinct comorbidities as columns and all patients as rows, with each table entry corresponding to 0 as the patient not having that diagnosis and 1 as the patient having that diagnosis. This table was then visualized as a UMAP with each point corresponding to one patient and encapsulating the similarity of that patient’s comorbidities to other patients based on distance. Correlations between variables (delirium status or sex) and UMAP coordinates were analyzed using Mann–Whitney *U*-tests. Differential comorbidity analysis between patients with delirium and controls was done using Fisher’s exact test and significance determined by Bonferroni-corrected threshold of *p*-value < 0.05. Analysis was done by generating a four-by-four matrix with each cohort group versus the number of patients with and without the diagnosis per group. Analysis was run using fisher.test function in R, and the odds ratio and *p*-values were extracted from the summary statistics. ICD10-CM blocks were also used to visualize differential comorbidity results. The OMOP concept table was used to map the SNOMED codes to ICD10 codes then mapped to categorical modules using the ICD10 codes. Overlaps between results of analyses using UCSF versus UC-wide datasets were done using hypergeometric test or Spearman correlation. Hypergeometric tests were done using the R function phyper (*q*−1, *m*, *n*, *k*), where *q* = total number of overlapping comorbidities between the two datasets, *m* = number of comorbidities significant in the UC-wide dataset, *n* = total number of comorbidities documented in both datasets, *k* = number of comorbidities significant in the UCSF dataset. A similar analysis was done for the sex-stratified analysis.

### Differential laboratory test results analysis

Laboratory test results were collected for tests done prior to the visit of interest, and the median values for all numerical lab tests were calculated. Lab tests that had more than 95% of patients missing results were excluded from the analysis, adapted from a prior study^21^. Patients with no value available for a lab test were excluded from the analysis. Lab value distributions were compared using Mann–Whitney *U*-tests across delirium status or sex.

### Longitudinal analyses

Time-to-event analysis was done by first identifying patients with a diagnosis of bipolar disorder (BD) and no prior delirium diagnosis. PS-matched control cohorts with no diagnosis of BD were identified using similar matching-criteria as described above. Time-to-event was calculated as time from first-time diagnosis of BD (or another non-BD diagnosis for the control patients) to either first inpatient delirium diagnosis, death, or loss to follow-up. Cox regression model was used to determine the hazard ratio, confidence intervals and significance (survival R^29^). Time-to-event analysis for mortality after delirium was done using a similar strategy.

### Statistics and reproducibility

All statistical analyses were performed using R Statistical Software version 4.3.2 (R Core Team 2023) and the specific statistical tests used for each experiment are outlined in detail above in each respective section as well as in the figure legends respectively.

### Reporting summary

Further information on research design is available in the Nature Portfolio Reporting Summary linked to this article.

## Results

### Identification of patients with a diagnosis of delirium and their matched controls

**Fig. 1:**
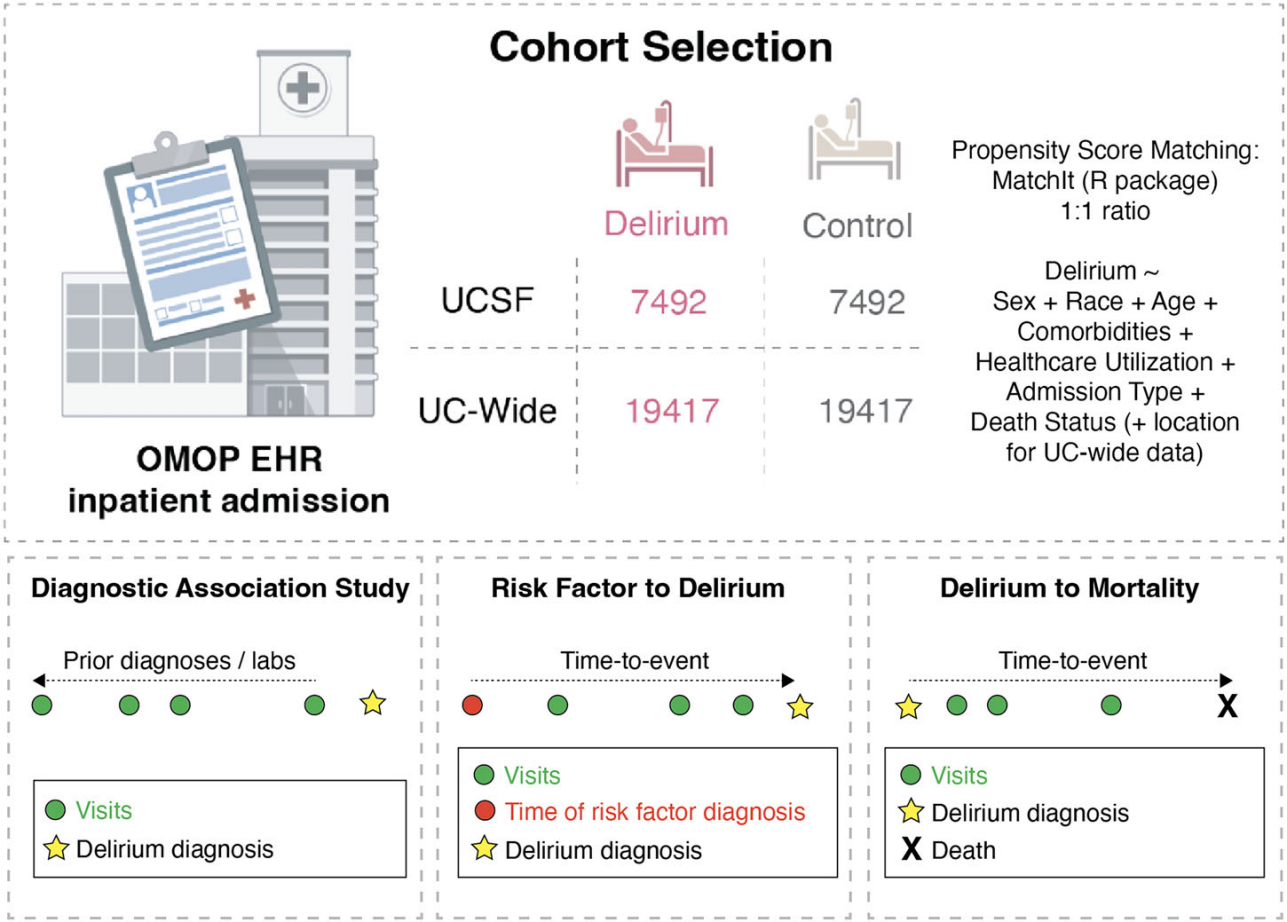
Schematic of study. Patient cohorts were selected from the OMOP electronic health record databases from UC San Francisco (UCSF) and UC-Wide databases (4 sites: UC Davis, UC San Diego, UC Los Angeles, UC Irvine). All patients and their first inpatient diagnosis of delirium were selected and a control cohort with no delirium diagnosis was selected using propensity score matching on the features listed in the formula. Association and longitudinal analyses were done using these cohorts. Association studies were done with prior diagnoses and prior laboratory results, with and without sex-stratification. Longitudinal analyses included time-to-delirium diagnosis after first-time diagnosis of selected potential diagnostic risk factor and time-to-mortality outcome after an inpatient delirium diagnosis. *n* = 7492 UCSF patients with or without a delirium diagnosis, *n* = 19,417 UC-wide patients with or without a delirium diagnosis. See also Supplementary Data 1. OMOP = Observational Medical Outcomes Partnership, UC = University of California.

We identified 7492 patients with an inpatient delirium diagnosis and 7492 propensity-score (PS)-matched control patients within the University of California, San Francisco (UCSF) de-identified electronic health record (EHR) database (~5 million patients total). Propensity score matching was used to create a matched case-control study as individual covariates may not be well-matched but the “propensity” to be either a case or control is well-matched, as illustrated in prior publications justifying this method^30,31^. Control patients were matched on the following demographic and inpatient visit features: age at admission, patient-identified race, sex, death during admission, length of the inpatient visit of interest in days, years of available EHR data, total number of inpatient visits prior to the visit of interest, total number of comorbidities prior to the visit of interest, and whether the visit of interest was in the ICU setting. The “visit of interest” corresponded to the visit where an inpatient delirium diagnosis was made for the delirium group or a randomly selected inpatient visit for the control group (Fig. 1). Similarly, a separate cohort of 19,417 patients with an inpatient delirium diagnosis and 19,417 PS-matched control cohort were identified from the UC-Wide EHR database (~8.6 million patients total, data from UC Davis, UC Los Angeles, UC Irvine, UC San Diego) with an additional matching criterion that included UC location (Fig. 1). These covariates were chosen based on prior EHR-based studies using PS-matched cohort selections with inclusion of additional covariates to control for health status and healthcare utilization frequency using indirect measures such as the total number of inpatient visits and comorbidities prior to the visit of interest and years of available EHR data^21,27,32,33^. Because patients who develop delirium tend to be at baseline more ill than those who do not develop delirium, we wanted to find a comparable control group that had similar baseline disease burden and identify what specific comorbidities are differentially enriched between patients who are similarly “ill”, but one group develops delirium and the other group does not.

Post-matching analysis showed adequate matching of covariates with similar PS distributions between the groups (Supplementary Fig. 1a, c) and absolute standardized mean differences <0.1, except for inpatient stay length which had a wide distribution in both databases (Supplementary Fig. 1b, d). A higher percentage of patients were male (UCSF 51.6%; UC-wide 56.5%) and Caucasian/White (UCSF 59.6%; UC-Wide 60.4%) with the average age at admission of interest being 63.9 years old in the UCSF dataset and 69.5 years old in the UC-wide dataset (Supplementary Data 1). Most of the visits were represented by non-ICU inpatient visits (UCSF 82.7%; UC-wide 67.6%) and the average stay length was 15.2 days in the UCSF dataset and 17.5 days in the UC-wide dataset, though there was a wide variation in the duration (Supplementary Data 1). Detailed demographic and inpatient visit features for the cohorts generated are shown in Supplementary Data 1. We defined inpatient delirium broadly using the Observational Medical Outcomes Partnership (OMOP) concept ID 373995 (corresponding to “Delirium”) and excluded diagnoses that had specific causes of delirium in the diagnosis name (such as alcohol-induced delirium or other substance-induced delirium). These exclusion criteria were used to focus on cases where a delirium diagnosis was made but the cause of the delirium was not readily known. Using this definition, we were able to capture 84% and 89% of all clinically recognized delirium-related visits in the UCSF and UC-wide databases, respectively, with delirium incidence within the wide range of published estimates for patients 65 years and older (9.5% mean, 8.9% SD in UCSF data; 3.5% mean, 1.9% SD in UC-wide data) (Supplementary Fig. 2a, b).

We used the Richmond agitation sedation scale (RASS) rating to corroborate the delirium diagnosis in the UC-wide data. RASS is a 10-point rating scale ranging from −5 to 5, with negative numbers corresponding to the level of sedation and positive numbers corresponding to the level of agitation. Though RASS is a more general measure of a patient’s level of sedation, validated mostly in the ICU setting, it has also been implemented more widely in the inpatient setting for detection of delirium^34,35^. Less than half of patients had documented RASS ratings during their visit of interest. Of the available ratings, patients with delirium had a statistically significant increase in the mean RASS rating compared to controls (Supplementary Fig. 2c), suggesting patients with a delirium diagnosis in this study were overall more agitated during the visit captured.

### Patients with delirium are more likely to be diagnosed with diseases of the nervous system, mental health, metabolic disorders, and infections compared to control patients

**Fig. 2:**
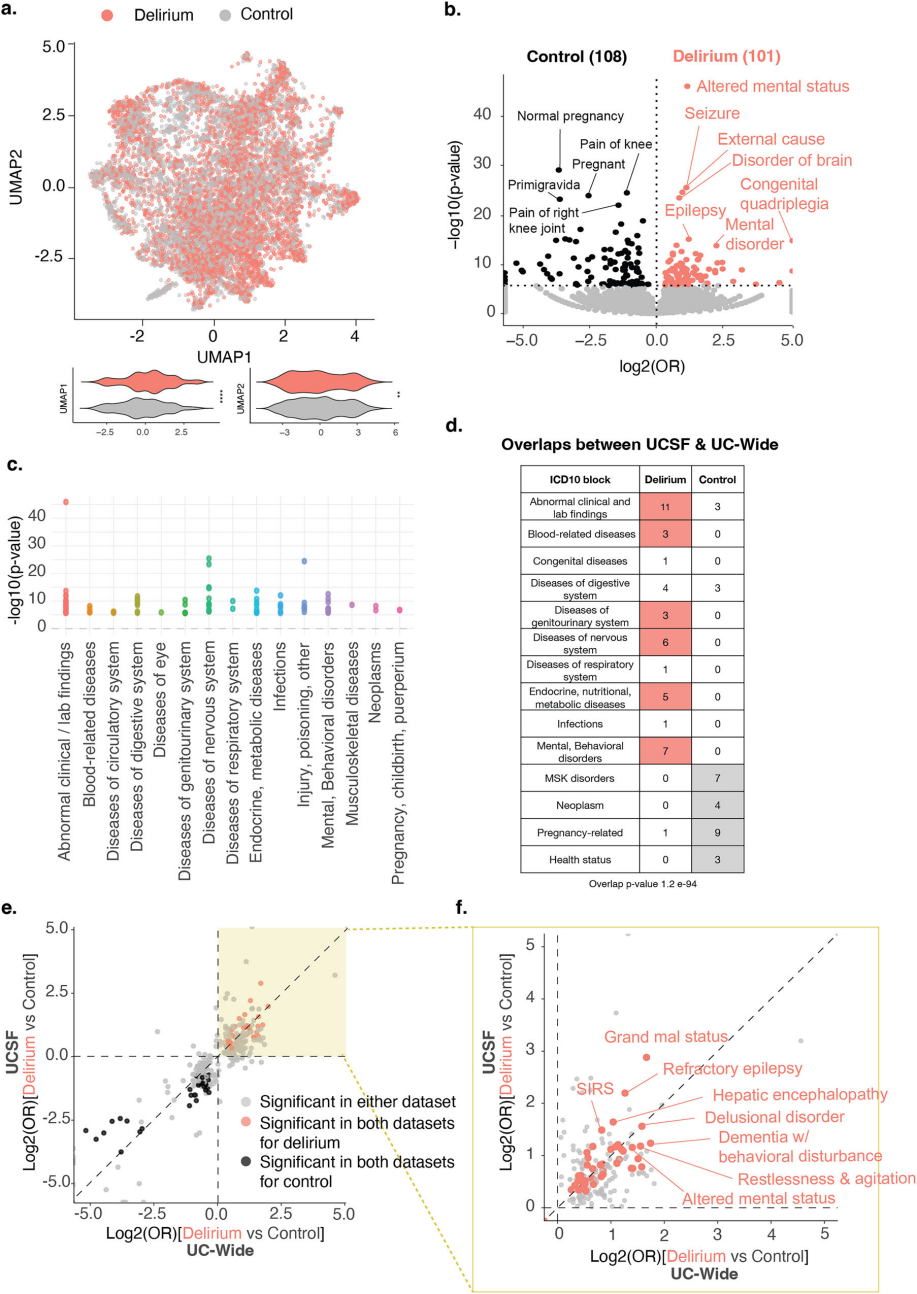
Diagnostic associations with delirium show enrichment of known risk factors of delirium. **a** UMAP representation of all first-time, non-delirium diagnoses prior to the inpatient visit of interest. Each dot represents a patient (salmon = 7492 patients with delirium, gray = 7492 control patients). Violin plots showing distribution of patients across UMAP component 1 (left) and 2 (right). *p*-values determined by two-sided Mann–Whitney *U*-tests. ****p-value < 2.2 × 10^−16^; **p-value 0.0086. **b** Volcano plot of differential comorbidities, with diagnoses enriched in controls in black (108 diagnoses) and in delirium patients in salmon (101 diagnoses) and non-significant diagnoses in gray. Significance determined by two-sided Fisher’s exact test with Bonferroni-corrected *p*-value  <  0.05 (at dotted horizontal line). OR = odds ratio. Most significant diagnoses highlighted by name. **c** ICD10-diagnostic block representation of significant differential comorbidities identified in **b** for patients with delirium. **d** Table showing number of diagnoses overlapping between UCSF and UC-Wide datasets in each ICD10 block for each patient group. Entries with at least 3 or more diagnoses in one patient group compared to the other are colored. Hypergeometric test used to evaluate overlap between the two datasets (*p*-value 1.2 × 10^−94^). **e** Log–log plot comparing differential diagnoses between UCSF and UC-Wide databases. Each dot represents a differential diagnosis that is significantly different between delirium versus control patients in either dataset (gray) or in both datasets (salmon for enrichment in delirium group, black for enrichment in control group) databases. Axis values represent log base 2 of the odds ratio between delirium versus control patients in the UC-Wide dataset on the *x*-axis and the UCSF dataset on the *y*-axis. Spearman correlation *ρ* = 0.94 when looking at points significant in both datasets. Dotted line represents perfect correlation between the magnitude of the odds ratios between the two datasets. **f** Zoomed in plot of the yellow-highlighted portion of plot in **e** to focus on the differential diagnoses significantly enriched in the delirium group, with gray representing diagnoses significant in either dataset and salmon representation diagnoses significant in both datasets. Diagnoses with the largest odds ratios are highlighted. See also Supplementary Fig. 3 and Supplementary Data 2-4. UMAP Uniform Manifold Approximation and Projection, EHR electronic health record, ICD10 International Classification of Diseases, 10th Revision, UC University of California, UCSF University of California San Francisco, MSK musculoskeletal, SIRS systemic inflammatory response syndrome.

To understand potential risk factors associated with an inpatient delirium diagnosis, we first collected all first-time diagnoses made during visits prior to the inpatient admission of interest, which did not include any diagnoses made during the inpatient admission of interest. Low-dimensional uniform manifold approximation and projection (UMAP) representation of all non-delirium diagnoses (19,590 features, SNOMED concept IDs) shows a statistically significant separation of patients with a delirium diagnosis versus matched controls by two-sided Mann–Whitney *U* test (UMAP 1, *p*-value < 2.2 × 10^−16^; UMAP 2 *p*-value 0.0086; Fig. 2a).

Differential association analyses of these comorbidities using Fisher’s exact test showed enrichment of distinct comorbidities for patients with delirium compared to control patients, with 101 diagnoses significantly enriched in patients with delirium versus 108 in controls, out of 19,583 diagnoses tested (Fig. 2b). Control patients had enrichment of diagnoses largely related to pregnancy and other health statuses as well as age-related musculoskeletal diagnoses such as pain in joints and osteoarthritis and skin findings such as melanocytic nevus (Fig. 2b, c, and Supplementary Data 2). Meanwhile, patients with delirium had enrichment of diagnoses related to diseases of the nervous system, including epilepsy and seizures, mental health and behavioral disorders, and acute diagnoses such as metabolic diseases and infections (Fig. 2b, c and Supplementary Data 2). Similar diagnostic associations were seen in the UC-wide cohort based on a hypergeometric test (*p*-value 1.2 × 10^−94^; Fig. 2d). Several comorbidities were significant in both datasets and had similar directionality in enrichment, though the magnitude of the odds ratios was distinct between datasets, adding more validity to these comorbidities given their consistencies between datasets (Fig. 2e, f, Supplementary Fig. 3, Supplementary Data 3, and 4). Categorizations of these overlapping diagnoses between the two databases by ICD10 diagnostic blocks showed enrichment of diagnoses related to certain disease categories in patients with delirium versus control patients, including blood-related diseases such as anemia, diseases of the genitourinary system such as urinary tract infections, diseases of the nervous system such as epilepsy, metabolic diseases such as hyponatremia, and mental health-related disorders such as bipolar disorder (Supplementary Fig. 3d and Supplementary Data 4). These findings are largely consistent with previously identified risk factors for delirium^3^.

### Differential laboratory findings corroborate differential associations between comorbidities and delirium

**Fig. 3:**
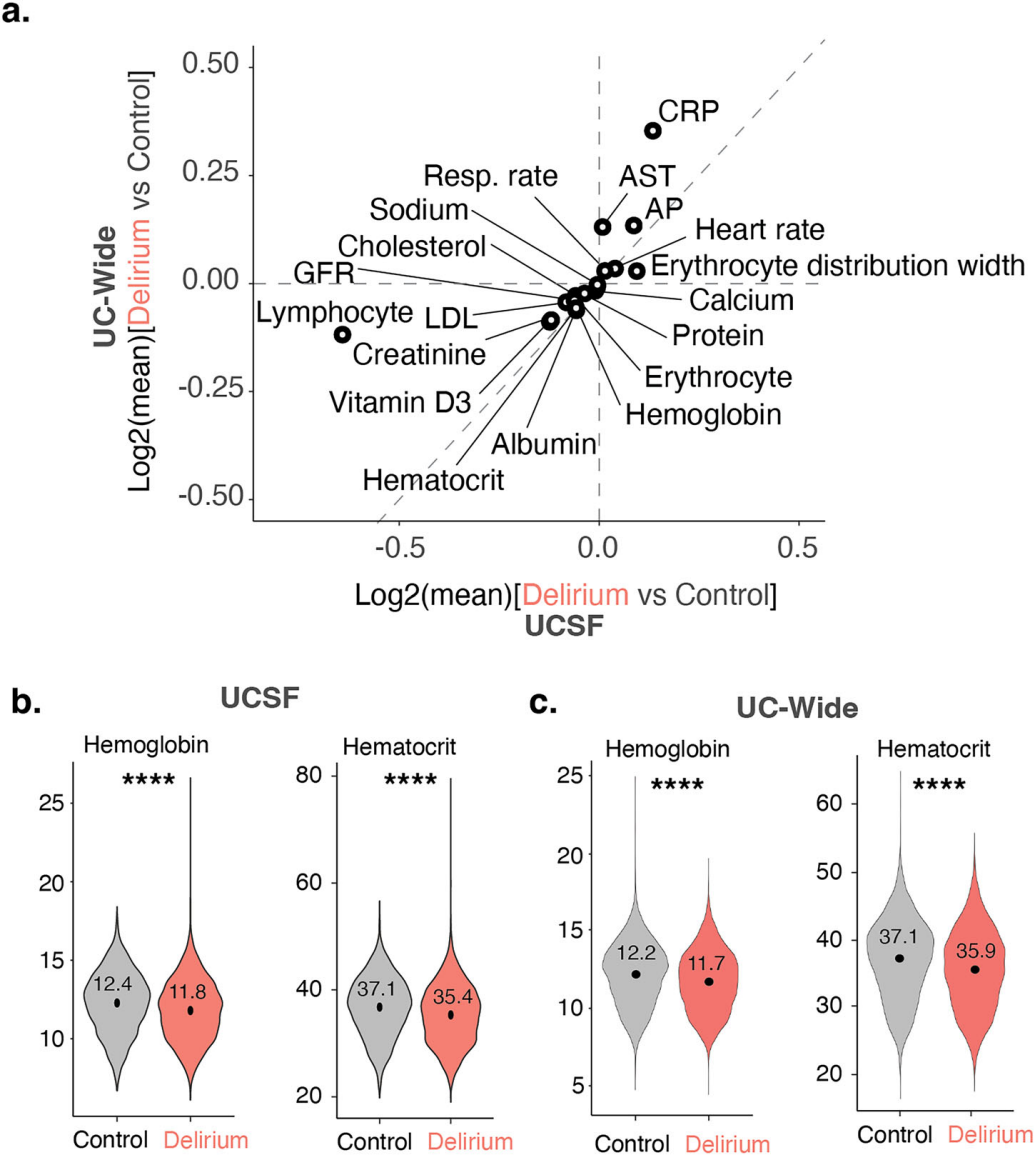
Identification of differential laboratory tests between delirium and control patients. **a** Log–log plot comparing all significantly differential laboratory results between delirium versus control patients in UCSF (*y*-axis) and UC-Wide (*x*-axis) databases. Axes reflect log base 2 of the median lab values for delirium versus control patients. Each dot represents a differential laboratory result that is significantly different between delirium versus control patients in both datasets. Dotted line represents perfect correlation between the magnitude of the odds ratios between the two datasets. **b** and **c** Violin plots showing distribution of patients and their median hemoglobin and hematocrit values for UCSF (**b**) and UC-wide (**c**) datasets. Black point denotes mean of population. For hemoglobin values: *n* = 5048 UCSF control patients; 4847 UCSF patients with delirium; 3346 UC-wide control patients; 2676 UC-wide patients with delirium. For hematocrit values: *n* = 5055 UCSF control patients; 4849 UCSF patients with delirium; 2748 UC-wide control patients; 2179 UC-wide patients with delirium. Two-sided Mann–Whitney *U*-test, ****p-value < 2.2 × 10^−16^. See also Supplementary Data 5. UC University of California, UCSF University of California San Francisco, AP alkaline phosphatase, AST aspartate aminotransferase, CRP C-reactive protein, GFR glomerular filtration rate, LDL low-density lipoprotein, Resp rate respiratory rate, SIRS systemic inflammatory response syndrome.

We also conducted enrichment analysis for median laboratory values and vital signs collected before the inpatient admission of interest. Patients with a delirium diagnosis had significantly higher median values of certain liver function tests, such as alkaline phosphatase and aspartate transferase, compared to controls in both UCSF and UC-wide datasets, suggesting potential liver dysfunction in these patients (Fig. 3a). Elevated median vital signs included heart rate and respiratory rate (Fig. 3a). Meanwhile, glomerular filtration rate and urine creatinine levels were decreased in patients with delirium compared to controls, consistent with kidney dysfunction (Fig. 3a). Hemoglobin, hematocrit, and erythrocyte counts were also significantly decreased in patients with delirium compared to controls, consistent with the association with an anemia diagnosis in these patients (Fig. 3). The UC-wide dataset also captured certain clinical test scores, including results from the Patient Health Questionnaire (PHQ)-2 and PHQ-9, consistent with the associations with depression in these patients (Supplementary Data 5).

### Sex-stratified analysis shows certain infections and dementia subtypes are sex-specific risk factors for delirium

To understand whether any of the comorbidities associated with delirium that we identified are sex-specific, we conducted a sex-stratified association analysis using the same cohort identified above (Supplementary Figs. 4 and 5). We identified several diagnoses that were significantly associated with delirium in only females, only males, or in both female and male patients with delirium compared to controls in both UCSF and UC-wide datasets (Supplementary Figs. 4c–e and 5c–e) as well as sex-specific laboratory results (Supplementary Figs. 4f and 5f).

**Fig. 4:**
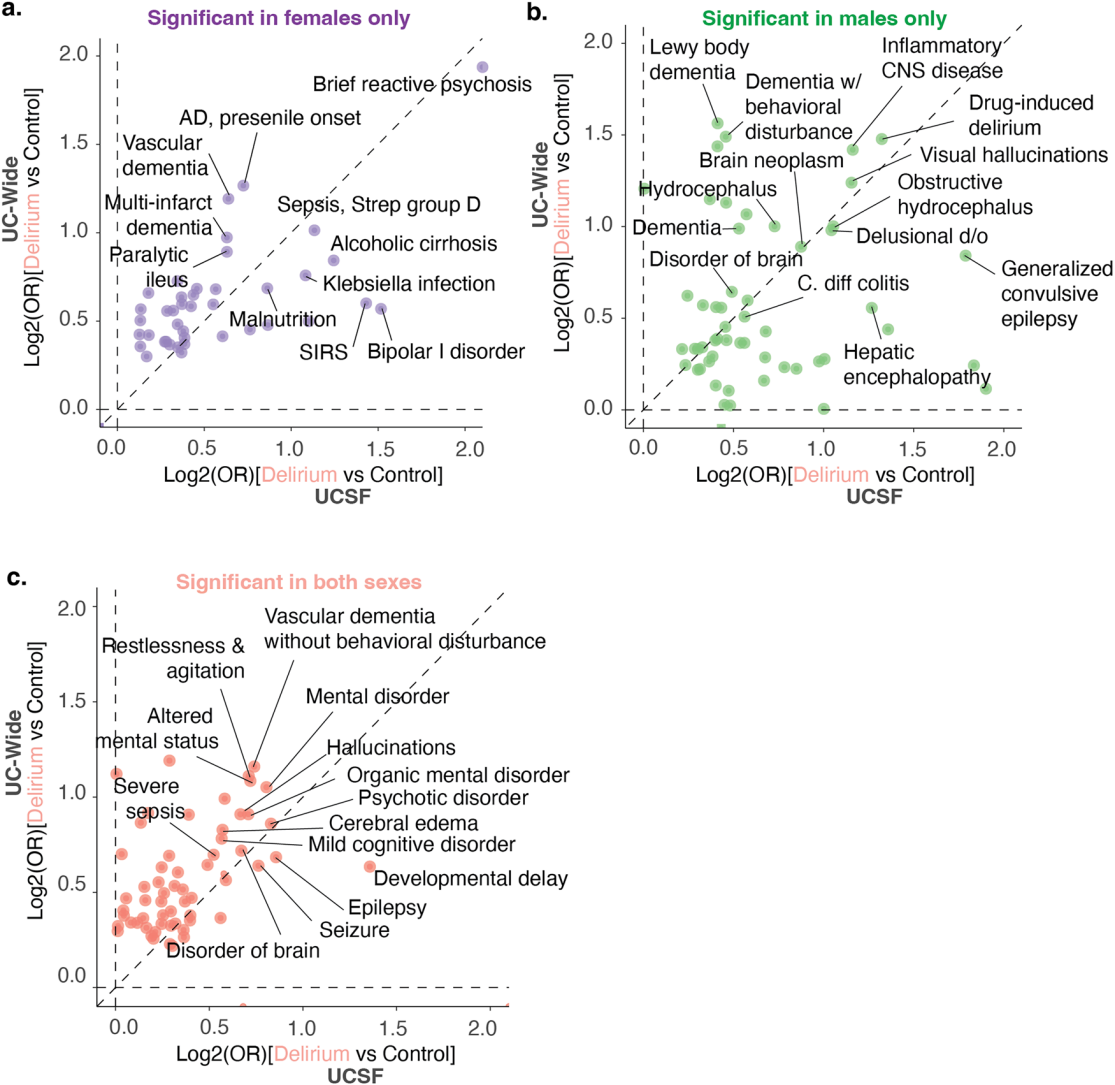
Identification of sex-specific diagnostic associations with delirium, including dementia subtypes and infections. Log–log plots comparing differential diagnoses enriched in delirium patients in UCSF (*x*-axis) versus UC -wide (*y*-axis) databases. Each dot represents a diagnosis that is significantly different between delirium versus control patients in both datasets. Axis values represent log base 2 of the odds ratio between delirium versus control patients in the UCSF dataset on the *x*-axis and the UC-Wide dataset on the *y*-axis. Plots split by diagnoses significant only in females (**a**), only in males (**b**), or in both (**c**). Diagnoses with largest OR values highlighted. Spearman correlation *ρ* = 0.9 (**a**), 0.35 (**b**), 0.55 (**c**). Dotted line represents perfect correlation between the magnitude of the odds ratios between the two datasets. For UCSF dataset: *n* = 3608 female control patients; 3637 female patients with delirium; 3836 male control patients; 3884 male patients with delirium. For UC-wide dataset: *n* = 8451 female control patients; 8458 female patients with delirium; 10,966 male control patients; 10,959 male patients with delirium. See also Supplementary Figs. 4 and 5 and Supplementary Data 6 and 7. OR odds ratio, UC University of California, UCSF University of California San Francisco, AD Alzheimer’s disease, *C. Diff* colitis *Clostridioides Difficile* colitis, CNS central nervous system, d/o disorder.

Diagnoses common to both male and female patients with delirium in both datasets were largely similar to the non-stratified analysis, including symptoms of delirium such as altered mental status, restlessness and agitation, and hallucinations, as well as known organic causes of delirium and altered mental status such as seizures, cognitive disorders and other mental disorders (Fig. 4c). Interestingly, female and male patients diagnosed with delirium had sex-specific associations with distinct infections and diseases of the nervous system that were statistically significant in both datasets. For instance, female patients with delirium had associations with encapsulated bacterial infections due to Streptococcal bacteria (OR UCSF 2.38; UC-wide 1.61), *Klebsiella pneumoniae* (OR UCSF 2.95, UC-wide 2.14)*, Escherichia coli* (OR UCSF 1.45, UC-wide 1.81), and enterococcal bacteria (OR UCSF 1.53, UC-wide 1.79), while males had associations with *Clostridioides difficile* infections (OR UCSF 3.89, UC-wide 1.55) (Fig. 4a, b). Sex-specific associations with subtypes of dementia were also seen, where females had significant associations with Alzheimer’s disease (OR UCSF 2.06, UC-wide 3.55) and vascular dementias with behavioral disturbances (OR UCSF 1.89, UC-wide 3.29) while males had associations with Diffuse Lewy Body disease (OR UCSF 1.51, UC-wide 4.22) and diagnoses related to symptoms of the disease such as visual hallucinations, falls, difficulty walking/muscle weakness, dysphagia, insomnia, and constipation (Fig. 4a, b, Supplementary Data 6, and 7). These sex-specific associations could be because these dementia subtypes are known to have sex-differences in their prevalence already^18^ or could point to sex-specific ways in which delirium manifest in different patient populations.

Though there were several sex-specific laboratory results found in the UCSF and UC-wide datasets, none were common between the two datasets. Laboratory findings associated with delirium in both male and female patients as well as between both datasets were similar to the results of the non-sex-stratified analysis, including decreased hemoglobin and hematocrit consistent with an anemia diagnosis, decreased GFR consistent with kidney dysfunction, elevated liver function tests such as alkaline phosphatase, and elevation of heart rate (Fig. 3a, Supplementary Figs. 4f and 5f).

### Longitudinal analysis from diagnosis of a potential risk factor of delirium to an inpatient delirium diagnosis validates comorbidity association study

**Fig. 5:**
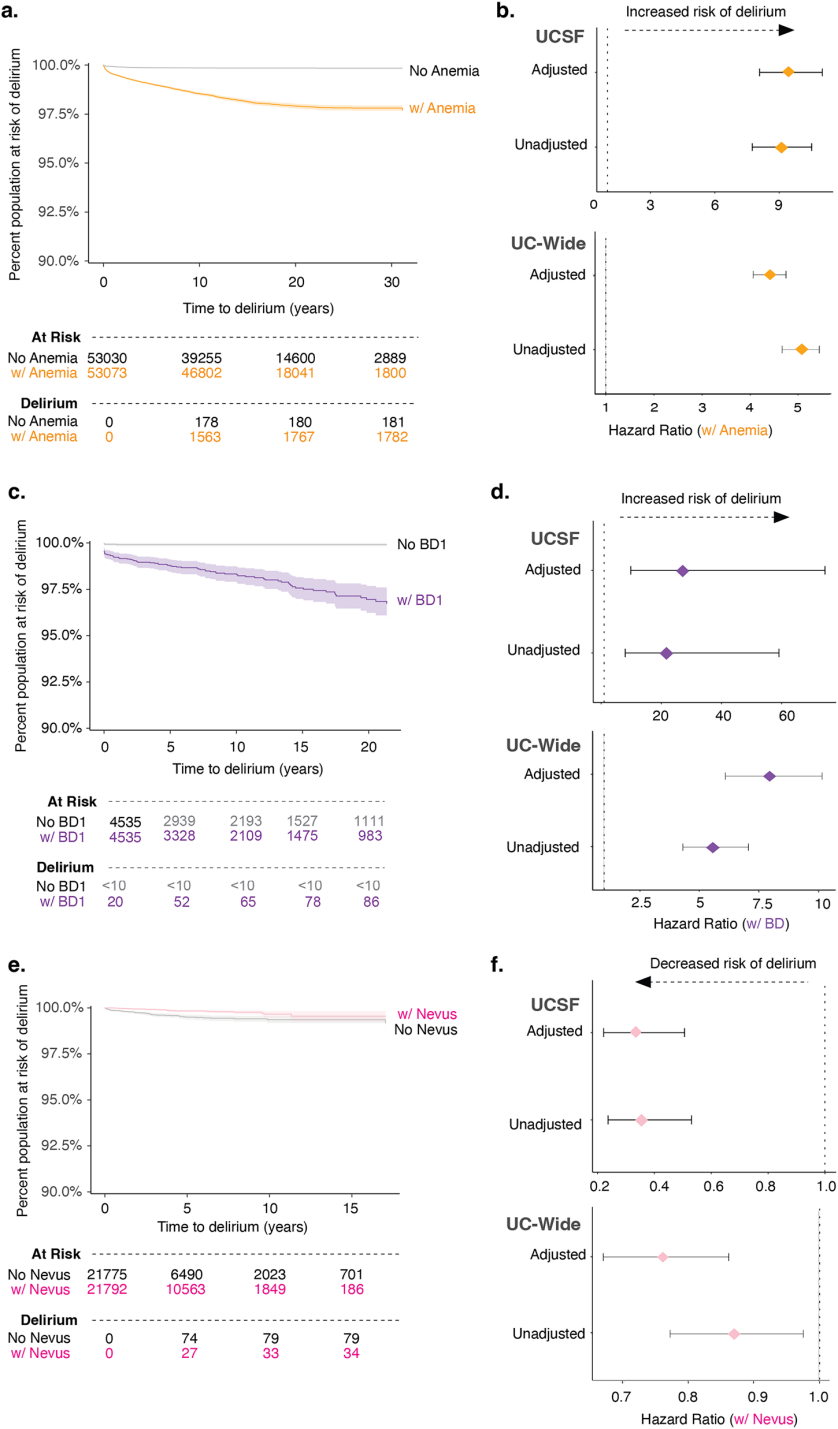
A prior diagnosis of anemia or bipolar disorder leads to increased risk of developing delirium. Kaplan–Meier curve showing time-to-event where event is defined as delirium, death, or loss to follow-up since the first-time diagnosis of anemia (**a**; *n* = 53,030 patients without anemia; 53,073 patients with anemia), bipolar disorder (**c**; *n* = 4535 patients without bipolar disorder; 4535 patients with bipolar disorder), and melanocytic nevus (**e**; *n* = 21,775 patients without melanocytic nevus; 21,792 patients with melanocytic nevus) in UCSF patients. Cox proportional hazard ratio analysis done for anemia (**b**), bipolar disorder (**d**), and melanocytic nevus (**f**) with unadjusted and adjusted analyses (adjusted for sex, age at admission, race, length of time in EHR, number of inpatient visits prior, total number of comorbidities, and length of follow-up time in EHR) in UCSF (top) and UC-wide (bottom) patients. Shaded areas of the Kaplan–Meier curves and error bars in forest plots represent 95% confidence intervals (see also Supplementary Figs. 6 and 7). UC University of California, UCSF University of California San Francisco, EHR electronic health record, BD bipolar disorder, W/ with.

To further understand several of the comorbidities associated with an inpatient delirium diagnosis identified above, we carried out a longitudinal time-to-event analysis for anemia and bipolar disorder. We chose these diagnoses for analysis given the higher association of blood-related disorders and mental and behavioral disorders in patients with delirium found through our association study (Fig. 2d). Patients with a first-time diagnosis of anemia and no prior diagnosis of delirium and a matched control group with no diagnosis of anemia and no prior diagnosis of delirium were identified. Control patients were matched on the following parameters: age at the visit of interest, patient-identified race, documented sex, years of available EHR data, total number of inpatient visits prior to the visit of interest, and total number of comorbidities prior to the visit of interest (Supplementary Fig. 6). Events were defined as admission for delirium, death, or loss to follow-up. Kaplan–Meier curve visualization of the data showed stratification by anemia diagnosis status, where those with anemia have increased probability of developing first-time inpatient delirium diagnosis (UCSF 3.4%, UC-wide 1.3% of anemia patients) than those without any anemia diagnosis (UCSF 0.3%, UC-wide 0.3% of control patients) over the course of ~30 years in the UCSF data and ~11 years in the UC-wide data (Fig. 5a and Supplementary Fig. 7a). Cox proportional hazard ratio analysis unadjusted and adjusted for demographics and visit features revealed a significant increased risk of delirium in those with an anemia diagnosis compared to controls (UCSF HR 9.4; 95% CI, 8.1–11; UC-wide HR 4.4; 95% CI, 4.1–4.7) (Fig. 5b). A similar analysis was done for patients with and without Bipolar I Disorder (BD1) in UCSF patients or bipolar disorder (unspecified type) in UC-wide patients. We found that a diagnosis of BD also increased risk of developing first-time inpatient delirium diagnosis (UCSF 1.9%, UC-wide 0.7% of BD patients) than those without a BD diagnosis (UCSF 0.01%, UC-wide 0.01% of control patients) with a HR of 27 (95% CI, 9.9–74.4) for UCSF patients and HR of 7.8 (95% CI, 6.0–10.0) for UC-wide patients over the course of ~20 years in the UCSF data and ~10 years in the UC-wide data (Fig. 5c, d, and Supplementary Fig. 7b).

Our association studies also found that certain diagnoses are more enriched in control patients compared to patients with delirium, including diagnoses under the ICD10-CM neoplasm category such as melanocytic nevus (Fig. 2d and Supplementary Data 4). We did a similar time-to-event analysis of patients with and without this diagnosis and found that, indeed, a prior diagnosis of melanocytic nevus modestly decreased the risk of developing delirium at a HR of 0.3 (95% CI, 0.2–0.5) for UCSF patients and HR of 0.76 (95% CI, 0.67–0.86) for UC-wide patients (Fig. 5e, f, and Supplementary Fig. 7c).

### A single inpatient delirium admission is associated with increased mortality

**Fig. 6:**
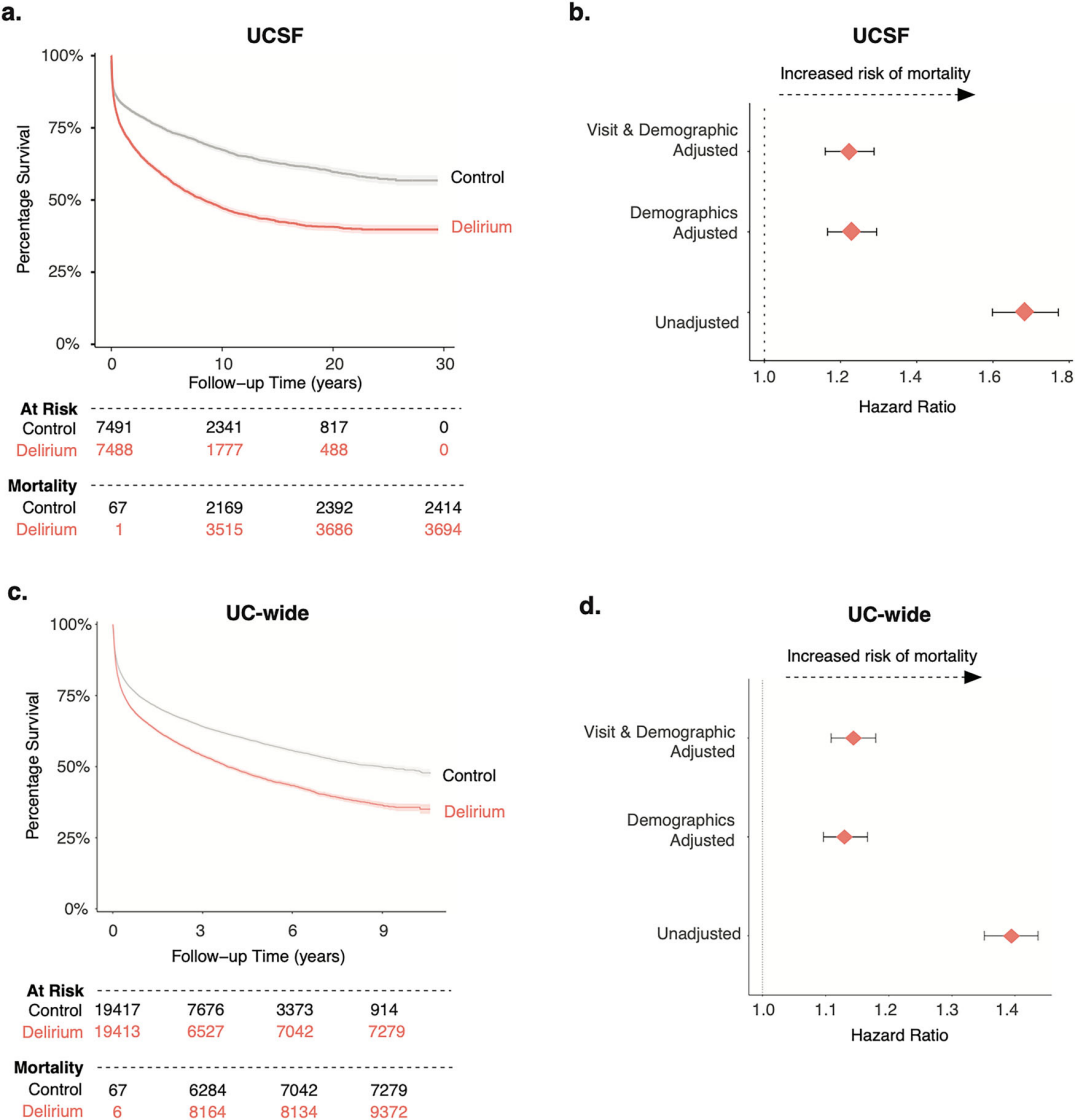
Increased mortality outcome after an inpatient delirium diagnosis. Kaplan–Meier survival curve showing time-to-death after inpatient admission of interest for control (gray) and delirium (salmon) patients in UCSF (**a**) and UC-Wide (**c**) data. Follow-up time periods differ between the two datasets given the discrepancy in the number of years captured by each dataset. Cox proportional hazard ratio analysis done for in UCSF (**b**) and UC-wide (**d**) cohort with unadjusted and adjusted analyses (adjusted for sex, age at admission, race, length of time in EHR, number of inpatient visits prior, total number of comorbidities, type of visit, visit length, and length of follow-up time in EHR). Shaded areas of the Kaplan–Meier curves and error bars in forest plots represent 95% confidence intervals. For UCSF dataset: *n* = 7491 control patients; 7488 patients with delirium. For UC-wide dataset: *n* = 19,417 control patients; 19,413 patients with delirium. EHR electronic health record, UC University of California, UCSF University of California San Francisco.

Previous meta-analyses have documented increased risk of mortality after an inpatient delirium admission^5^. To validate these findings in a larger population size while also accounting for more covariates than previously tested, including health status, we used our cohort of patients with delirium and their matched controls to conduct a longitudinal time-to-event analysis, where an event was defined as mortality or loss to follow-up. Kaplan–Meier survival curve visualization of the data showed different probabilities of survival rates between patients with an inpatient delirium admission (median 8.47 years; 95% CI, 7.8–9.35 in delirium group) versus control patients (Fig. 6a). Cox proportional hazard ratio analysis unadjusted and adjusted for demographic characteristics (sex, age at admission, race, length of time in EHR, number of inpatient visits prior, total number of comorbidities) and visit features (type of visit, visit length, and length of follow-up time in EHR) revealed a significant increased risk of death in those with a delirium diagnosis compared to controls (HR 1.2; 95% CI, 1.16–1.29) (Fig. 6b). A similar analysis for the UC-wide patient cohort also showed increased mortality in those with a delirium diagnosis compared to controls with a HR of 1.14 (95% CI, 1.1–1.18) (median 8.96 years; 95% CI, 8.16–10.1 in control group; median 3.83 years; 95% CI, 3.71–4.1 in delirium group) (Fig. 6c, d).

## Discussion

In the past few decades, clinical data from the EHR combined with integrative computational approaches have enabled the dissection of complex, heterogeneous diseases in an unbiased manner. This study aims to apply this strategy to better understand both risk factors and outcomes of patients who are diagnosed with delirium while in the hospital. As expected, we found that many diagnoses related to delirium (such as “mental health” or “brain disorders”) or related to the symptom of delirium (such as “restlessness and agitation” or “altered mental status”) as well as well-known risk factors of delirium emerged through our analysis looking at comorbidities associated with an inpatient delirium diagnosis, including chronic neurological conditions such as dementia and epilepsy as well as acute conditions such as infections and metabolic disturbances. These findings are expected based on prior publications and point to the consistency and internal validity of our approach^3,36,37^. Our sex-stratified association studies revealed that, even within these large categories of diseases of the nervous system and infections, subtypes of conditions were sex-specific. We also validated several of these comorbidities associated with delirium, including anemia and bipolar disorder, through time-to-event analyses. Finally, we showed that a delirium diagnosis is independently associated with an increased risk of mortality, with UCSF and UC-wide data showing a median survival of ~8.5 and ~4 years, respectively, for patients with one inpatient delirium diagnosis.

To our knowledge, this is the first study to conduct a deep analysis of delirium versus control patients using the EHR data with over 7000 patients with delirium in the exploratory dataset and ~20,000 patients in the validation dataset. We aimed to find an appropriately matched control cohort by using propensity score matching and including covariates that assess patient health status as well as frequency of healthcare utilization. We also did not exclude patients based on prior cutoffs such as age thresholds or ICU versus non-ICU admissions, as done by previous studies^5–7,15^. This strategy enabled us to include as many delirium cases as possible with inclusion of as many covariates as observable through the EHR. One of the strengths of this study was the finding that a delirium diagnosis is independently associated with an increased risk of mortality even after adjustments of important covariates. Prior studies have found inconsistent results, some reporting a significant increased association with mortality, while others have not^5,8–10^. Those that have found a significant association were mostly meta-analyses conducted in critically ill patients or focused on patients older than 65 years old^38,39^. These discrepancies may be related to differences in clinical settings, patient populations, limited sample sizes, and confounding variables. Our study aimed to address these limitations from previous studies by leveraging a large sample size, adjusting for as many important covariates as possible, and the inclusion of all patient population ages and clinical settings. The hazard ratios found in our studies generally agree with prior studies that have shown an association between delirium and mortality (HR 1.95^5^, adjusted RR 1.5-5.4 in medical, non-intensive care settings^37^).

Even when the cohorts were matched based on the number of comorbidities, we were able to find comorbidities differentially enriched in patients with versus without delirium, that may help with risk prediction. For instance, anemia before admission for delirium was enriched in delirium patients compared to controls in both UCSF and UC-wide datasets. This was also confirmed at the laboratory level, with significant median hemoglobin difference in patients with and without delirium (11.8 versus 12.4, respectively), though these values and the absolute difference in the median values are likely not clinically meaningful. Our longitudinal analysis validated our comorbidity analysis and provided an understanding of the time between diagnosis of a risk factor to the development of delirium. In the UCSF data, for instance, roughly 3% of patients with anemia went on to develop delirium within 10 years of the diagnosis while less than 0.5% of patients without anemia developed delirium in this timeframe (hazard ratio of 9.4). One study in 700 patients specifically undergoing lumbar spinal fusion found that perioperative anemia is a risk factor for developing delirium post-operation^40^. Our study confirms that a diagnosis of anemia may be a more generalizable risk factor for developing inpatient delirium^41^, though ours is the first study to demonstrate through longitudinal studies that anemia could be a marker of later risk, even years after, of developing delirium. Further studies need to be done to understand whether early detection and treatment of anemia proactively could reduce subsequent delirium^42^.

The large number of patients also enabled us to dissect the diagnostic associations with delirium in a sex-stratified manner. Our findings point to potentially new biological insights into the pathophysiology of delirium that are sex dependent. For instance, male patients had a significant association with *Clostridioides difficile* infections, an infection that often develops after antibiotic use. This could point to either the *Clostridioides difficile* toxin and the downstream consequences of the infection being associated with delirium or the antibiotic that was used that led to the infection being associated with delirium. Meanwhile, *Klebsiella* and *E. coli* infection were enriched in female patients with delirium, organisms that most commonly cause urinary tract infections, a source of infection more common in females compared to males. Dementia, one of the known risk factors of delirium, is also known to have sex-biased features, including in prevalence, clinical progression, and neuropathological findings^18^. Interestingly, our association studies found that subtypes of dementia were associated with delirium in a sex-dependent manner. The caveat to these diagnoses is that there may be baseline biases of ordering certain tests for one sex versus another (for example, urinalyses obtained more frequently in female patients to assess urinary tract infections compared to male patients) as well as sex difference in the rates/prevalence of certain diseases and diagnoses. Further studies will need to be done to understand whether this is due to these underlying biases or underlying sex differences in the prevalence of these dementia subtypes or sex differences in the pathophysiology of these dementias contributing to delirium.

There are several limitations to our study that need further investigation. In general, the use of EHR data has inherent limitations such as not capturing all the covariates that may be relevant or the data being biased towards what is actually documented in the EHR based on clinical judgment and recognition. We have tried to address these concerns by using stringent matching protocols and including several covariates in the model for matching to find a balanced cohort. We also matched on the time of diagnosis and follow up time period as a way to account for immortal time biases, as recommended by a previously published study using EHR data^43^. Given that illness-severity is a well-studied risk factor for delirium, we used several indirect metrics of health status, including number of inpatient admissions and number of comorbidities, to find an appropriately matched control cohort to our delirium cohort. In this way, we were able to compare groups with similar health statuses to extract more subtle risk factors that may be associated with delirium. Though we matched on the number of comorbidities to identify a control cohort with a similar health status, some comorbidities are more functionally debilitating and severe than others. Not all factors relating to delirium, or any condition of interest are captured or measured within the EHR as the data reflects only what is assessed or entered by clinicians. Given our control group had more pregnancy-related diagnoses, this suggests that though the control group may have a similar frequency of healthcare utilization, they are overall healthier than the matched delirium cohort. Future studies could use other health status metrics, such as the Charlson Comorbidity Index, which was not available in the datasets we used, or the American Society of Anesthesiologists (ASA) physical status classification score^44^. ASA was available in our UC-wide dataset and was significantly higher in patients with delirium compared to control patients (Data not shown). The median values, however, were 2.95 in control patients versus 3.05 in delirium patients, suggesting this difference is likely not clinically meaningful, given the scores are integer numbers.

Defining an accurate delirium diagnosis in the EHR is challenging. Detection and clinical diagnosis of delirium itself is often challenging and likely underdiagnosed^3,45^. As a result, the published prevalence of delirium in older patients varies widely, with one meta-analysis finding the average prevalence to be 23%, though estimates ranged from 4% to 54%^4^, while another study found prevalence to be as high as 88% in palliative care patients^46^, and the incidence estimate also varying widely from 2% in general medical settings to 82% in intensive care settings^2,37,47^. Variability is likely due to variations in the clinical diagnosis of delirium as well as differences in practice settings and the patient populations captured. This is also seen in our study where the incidence of delirium diagnoses are different between the two datasets used (Supplementary Fig. 2a, b), which could be due to capturing different clinical settings as the UC-Wide dataset had more ICU visits captured compared to the UCSF dataset (31.6% versus 7.6%, respectively), and may also point to practice differences in clinical monitoring and diagnosing of delirium within each hospital system, and likely not a true epidemiological difference. This difference between the datasets would likely lead to under detection of delirium in the UC-wide dataset, leading to fewer differential risk factors between control patients and patients with delirium. By focusing on the risk factors and outcomes that were commonly found between the two datasets, we were able to add more confidence to these findings.

Documentation of delirium is unfortunately inconsistent in the EHR even with a confirmed diagnosis, with one study finding that only 9 out of 25 cases coded by ICD-9 code^48^. Further, by using diagnostic-coding as our way of capturing delirium biases us to cases that are clinically recognized and therefore more likely to exclude subtypes of delirium that are harder to detect such as hypoactive delirium. RASS scores were available for some of our patients in the UC-wide dataset and showed scores were different between patients with delirium and controls, with RASS scores being more positive in patients with delirium, suggesting a bias in capturing hyperactive delirium or clinically recognized delirium. We, however, did not use RASS as a way to include or exclude patient as it is not a tool specific to delirium but rather an overall metric of sedation status. Moreover, our association with bipolar I disorder and its elevated longitudinal risk of delirium could be due to mis-diagnosis of a manic episode as delirium, confounding these results. Future studies could include more patients by identifying delirium symptoms in clinical notes or including patients who have been screened for delirium using clinical tools such as the 4A’s Test (4AT) or the confusion assessment method (CAM)^49,50^, which were not available in the datasets we used. More consistent and reliable capturing of delirium diagnoses in the EHR could facilitate future studies better characterizing the different subtypes of delirium as well as focus on differential risk factors and outcome analyses based on subtypes.

In this study, the definition of sex in the EHR is likely a combination of sex assigned at birth, legal sex, and sex determined by the clinician. Documentation of gender identity remains a challenge in the EHR and only recently has garnered attention to provide more inclusive and affirmative health care for all patients^51,52^. Further studies will need to be done to understand whether the associations found in this study are different when taking into context biological sex versus patient-reported gender identity. Similarly, other social determinants of health were not taken into account in this study, such as the patient’s level of formal education, which can influence cognitive reserve, as well as family/caregiver dynamics.

Overall, our study demonstrates the powerful application of the EHR to study heterogeneous disease processes and to better understand the risk factors and outcomes of disease at both a large patient population scale and a longitudinal time course. These results not only confirmed some of the known risk factors of delirium but also generated several new clinical hypotheses that will need to be further investigated. Our large sample size and inclusions of many important covariates also enabled a clear and significant association between delirium and poor mortality outcomes. Applications of these analyses on other data types such as medication history may also expand the potential risk factors for delirium and increase the predictive power for the condition. These findings could also help develop future modeling studies for predicting which patients will develop delirium to focus prevention efforts on these patients. Understanding the impact of sex differences in delirium remains understudied, and this study points to the importance of doing sex-stratified analyses and the potentially interesting pathophysiology of delirium that interacts with sex.

## Supplementary information

Supplementary Information

Description of Additional Supplementary Files

Supplementary Data 1

Supplementary Data 2

Supplementary Data 3

Supplementary Data 4

Supplementary Data 5

Supplementary Data 6

Supplementary Data 7

Reporting Summary

## Data Availability

The UCSF EHR database is available to individuals affiliated with UCSF who can contact the UCSF’s Clinical and Translational Science Institute (CTSI) (ctsi@ucsf.edu) or the UCSF’s Information Commons team for more information (Info.Commons@ucsf.edu). The UC-wide EHR database is only available to UC researchers who have completed analyses in their respective UC first and have provided justification for scaling their analyses across UC health centers (more details at https://www.ucop.edu/uc-health/departments/center-for-data-driven-insights-and-innovations-cdi2.html or by contacting healthdata@ucop.edu. The source data for Fig. 2 can be found in Supplementary Data 2–4, for Fig. 3 in Supplementary Data 5, for Fig. 4 in Supplementary Data 6 and 7. We are unable to share individual-level data, even in de-identified form, due to the terms of our University of California Health Data Warehouse (UCHDW) Data Use Agreement. The agreement explicitly prohibits the transfer of data to individuals or entities outside the University of California system and restricts access and use to approved purposes under strict privacy and security policies. These safeguards are in place to ensure compliance with federal and state laws, including HIPAA and CMIA. This study did not generate new unique reagents. Further information and requests for resources and reagents should be directed to and will be fulfilled by the lead contact, Marina Sirota (marina.sirota@ucsf.edu).

## Code availability

The code used for analysis is available on GitHub at https://github.com/kozlama/delirium_EHR (10.5281/zenodo.15666149)^53^. Any additional information required to reanalyze the data reported in this paper is available from the lead contact upon request.
