# Supplementary material for: Comorbidities associated with a clinically-recognized delirium diagnosis in the hospital using real world data
Source: Commun Med (Lond). 2025 Jul 22;5:304. doi: 10.1038/s43856-025-00986-5 (PMC12284072; doi:10.1038/s43856-025-00986-5)
Supplement: Supplementary file 1 — Supplementary Information [file 43856_2025_986_MOESM1_ESM.pdf]

Supplementary Figure 1 (Related to Figure 1)
Matching results of delirium and control cohort from UCSF and UC-Wide EHR datasets

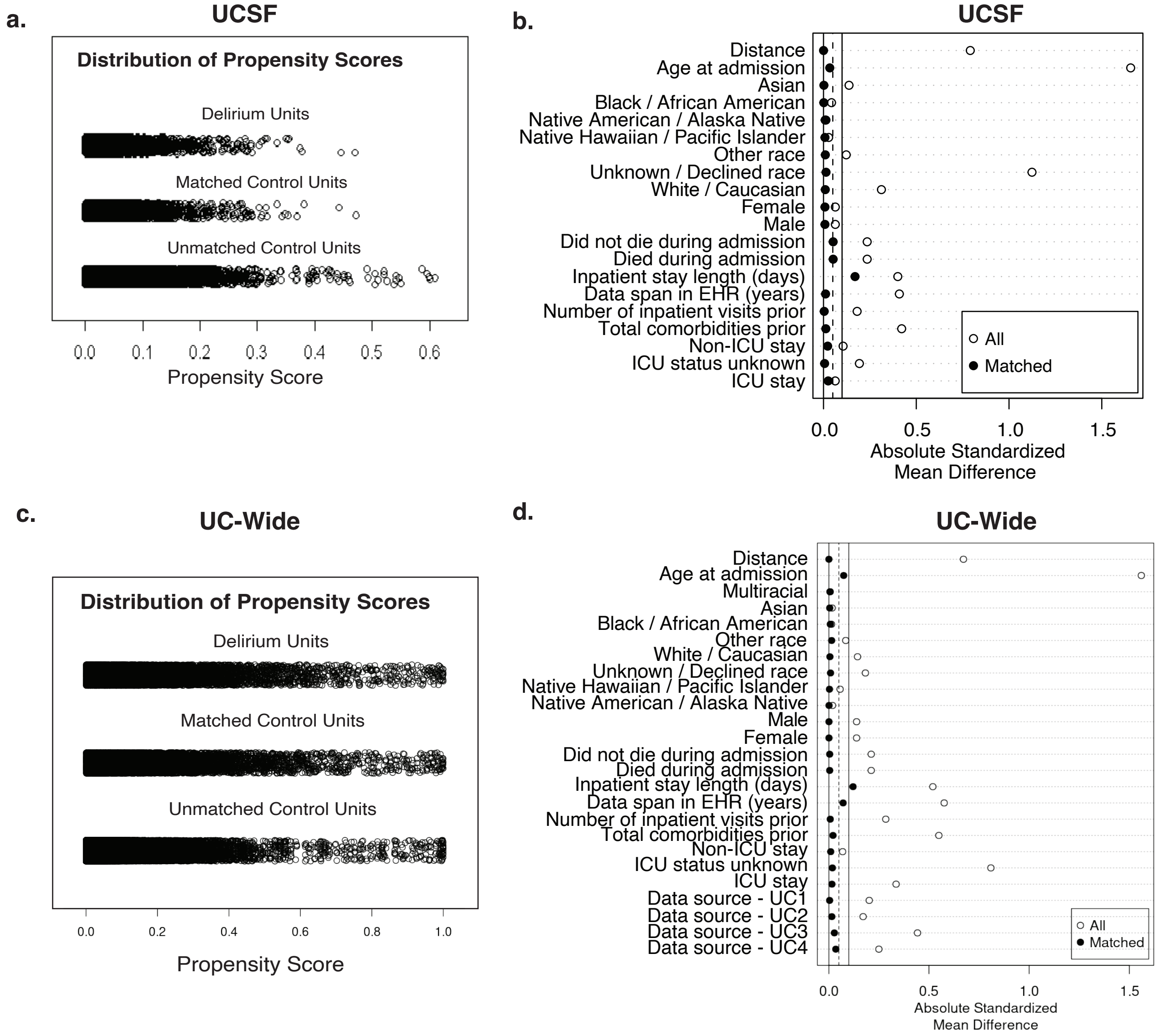

a-d. Distribution of propensity scores for patients with delirium, matched controls, and unmatched controls for UCSF (a) and UC-wide data (c). Absolute standardized mean difference between matched patients (black dot) versus all patients from the selection pool (white dot) for each demographic parameter used in the matching for UCSF (b) and UC-wide data (d).

Supplementary Figure 2 (Related to Figure 1)
Delirium prevalence in UCSF and UC-wide EHR

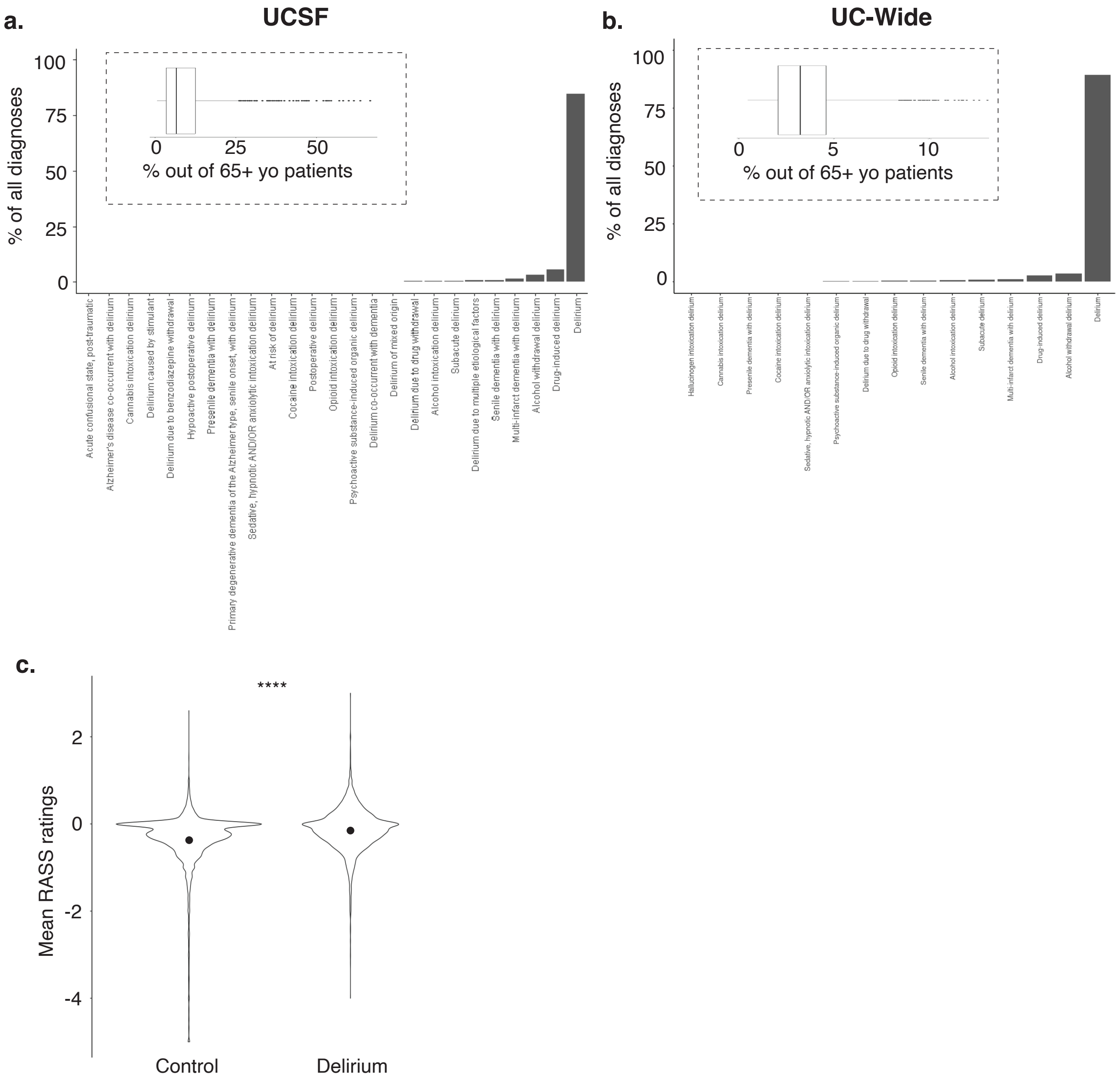

a-b. Bar plot of the percent of diagnoses out of all delirium-related diagnoses in UCSF (a) and UC-wide data (b). Inset boxplot showing percent of patients with a delirium diagnosis out of all patients admitted to the hospital on a given visit date who are 65 years or older at UCSF (a) and UC-wide (b). c. Violin plots of mean RASS ratings during the visit of interest for control versus patients with delirium in the UC-wide data. Significance based on Student's t-test, p-value < 2.2e-16.

Supplementary Figure 3 (related to Figure 2): Diagnostic associations with delirium in UC-wide data

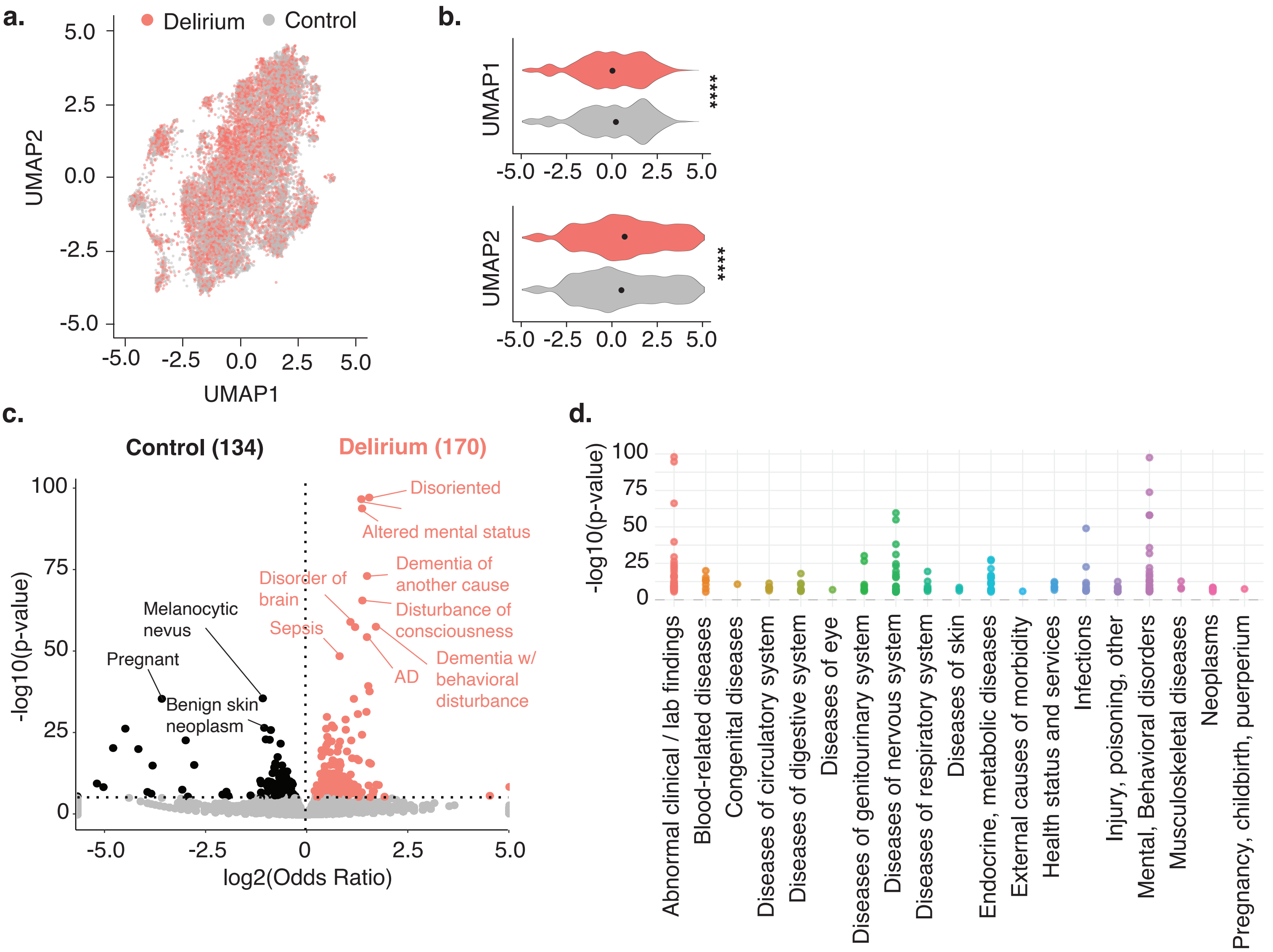

a.UMAP representation of all first-time, non-delirium diagnoses prior to the inpatient visit of interest. Each dot represents a patient (salmon = patient with delirium, grey = control patient). b. Violin plots showing distribution of patients across UMAP component 1 (top) and 2 (bottom). p-values determined by two-sided Mann–Whitney U-test. **** = p-value 1.5e-12; **** = p-value < 2.2e-16. c. Volcano plot of differential comorbidities, with diagnoses enriched in controls in black (134 diagnoses) and in delirium patients in salmon (170 diagnoses) and non-significant diagnoses in grey. Significance determined by two-sided Fisher's exact test with Bonferroni-corrected p-value < 0.05 (at dotted horizontal line). OR = odds ratio. Most significant diagnoses highlighted by name. d. ICD10-diagnostic block representation of significant differential comorbidities identified in (c) for patients with delirium.

Supplemental Figure 4 (related to Fig 4):
Diagnostic and laboratory associations with delirium by sex in UCSF data

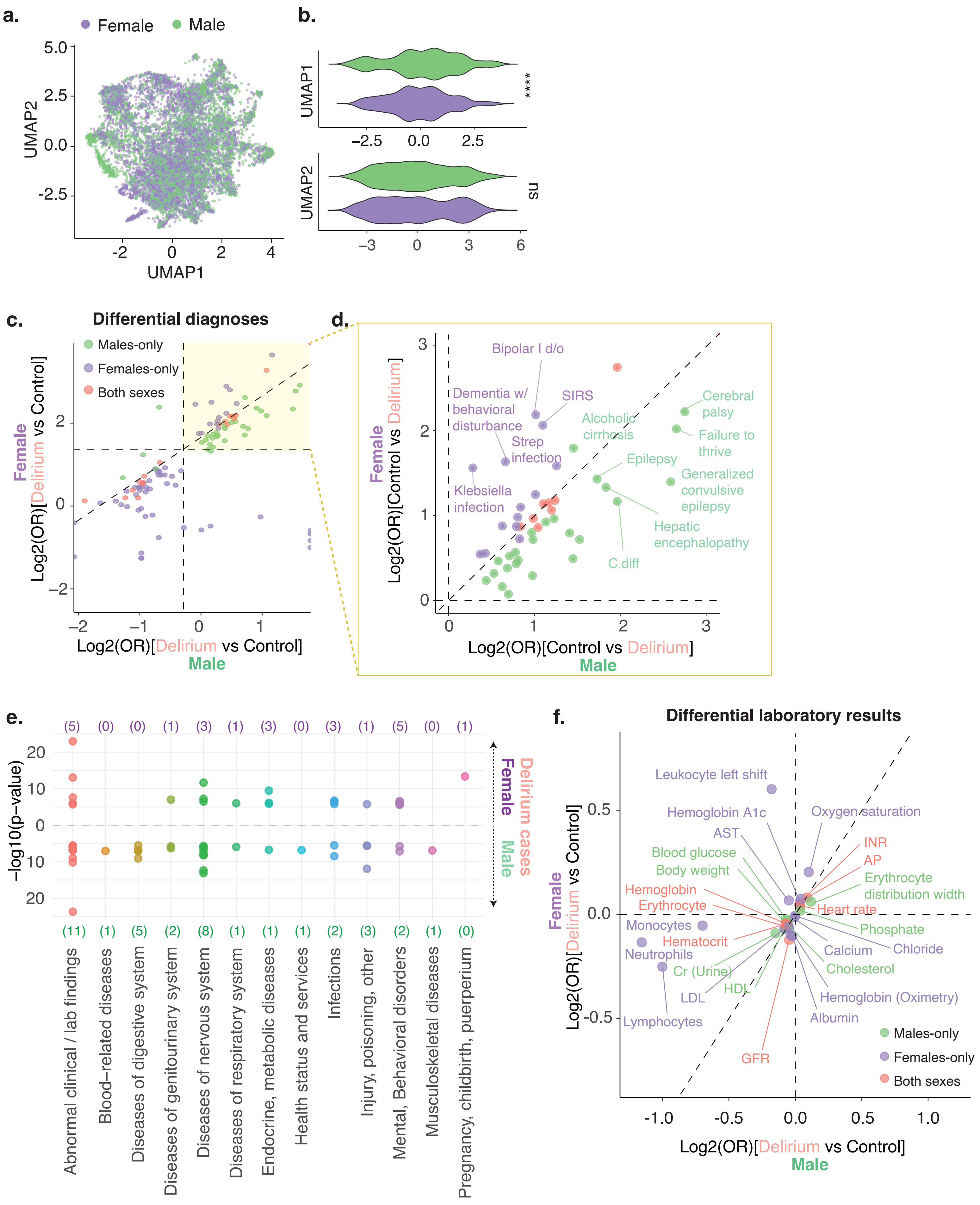

a.UMAP representation of all first-time, non-delirium diagnoses prior to the inpatient visit of interest. Each dot represents a patient (purple = female, green = male). b. Violin plots showing distribution of patients across UMAP principle component 1 (top) and 2 (bottom). p-values determined by two-sided Mann–Whitney U-test. **** = p-value < 2.2e-16. c. Log-log plot comparing differential diagnoses between female and male patients. Diagnoses significant in females only (purple), males only (green), or in both sexes (pink). d. Zoomed in plot of the yellow-highlighted portion of plot in (c). Significant comorbidities found in either male or female patients highlighted. e. ICD10-diagnostic block representation of significant differential comorbidities identified in (c) for patients with delirium, female (top), male (bottom). f. Log-log plot comparing differential laboratory results between female and male patients. Laboratory tests significant in females only (purple), males only (green), or in both sexes (pink).

Supplemental Figure 5 (related to Fig 4):
Diagnostic and laboratory associations with delirium by sex in UC-wide data

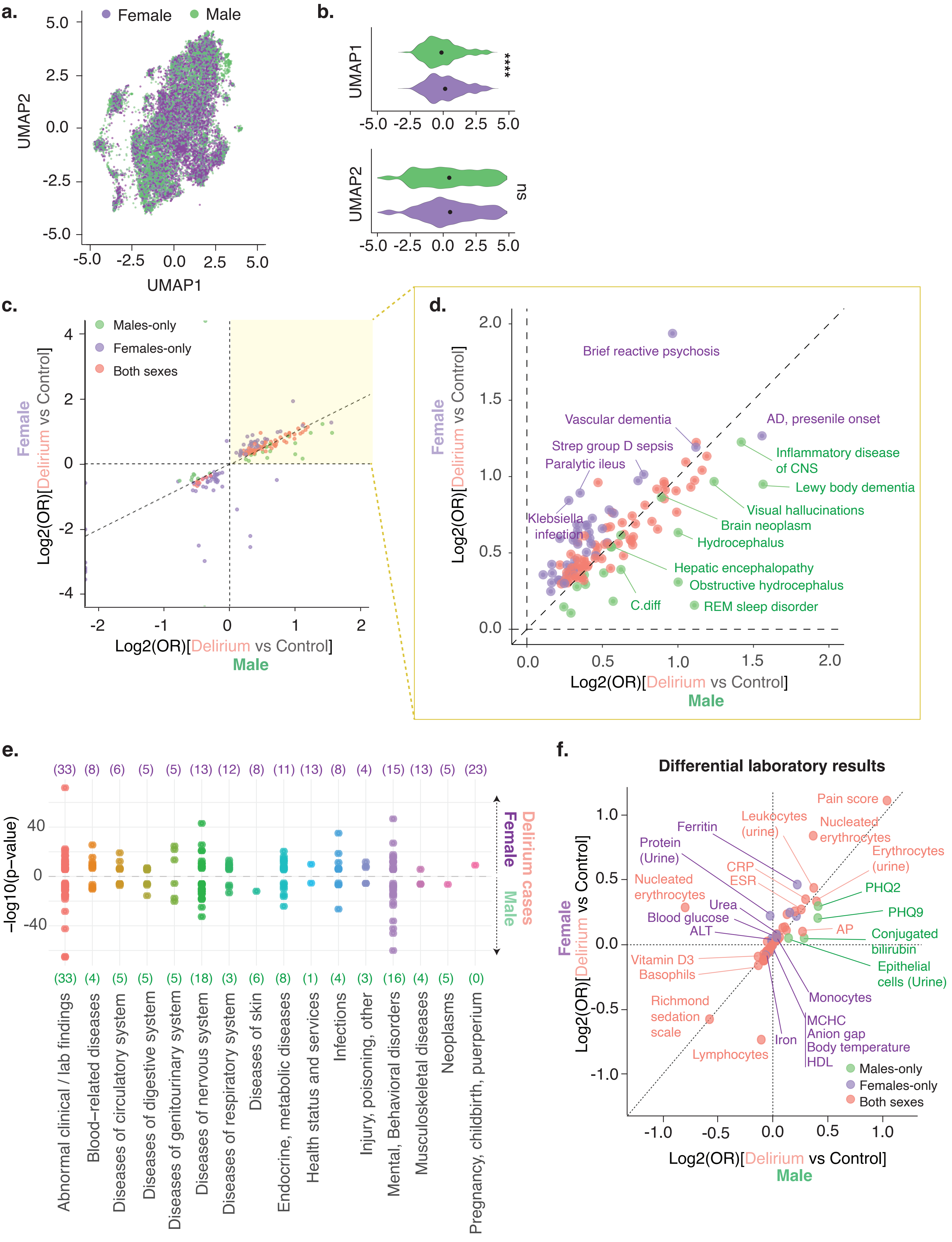

a.UMAP representation of all first-time, non-delirium diagnoses prior to the inpatient visit of interest. Each dot represents a patient (purple = female, green = male). b. Violin plots showing distribution of patients across UMAP principle component 1 (top) and 2 (bottom). p-values determined by two-sided Mann–Whitney U-test. **** = p-value = 5.3e-15. c. Log-log plot comparing differential diagnoses between female and male patients. Diagnoses significant in females only (purple), males only (green), or in both sexes (pink). d. Zoomed in plot of the yellow-highlighted portion of plot in (c). Significant comorbidities found in either male or female patients highlighted. e. ICD10-diagnostic block representation of significant differential comorbidities identified in (c) for patients with delirium, female (top), male (bottom). f. Log-log plot comparing differential laboratory results between female and male patients. Laboratory tests significant in females only (purple), males only (green), or in both sexes (pink).

Supplementary Figure 6 (Related to Figure 5)
Matching results of delirium and control cohort from UCSF and UC-Wide EHR datasets

a.

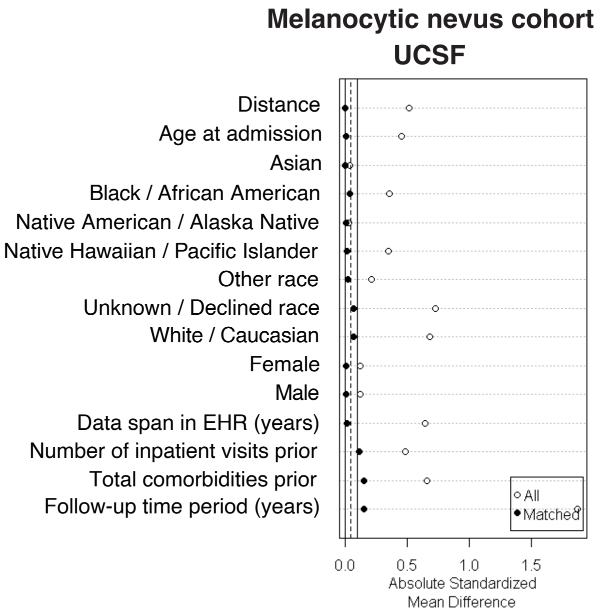

b.

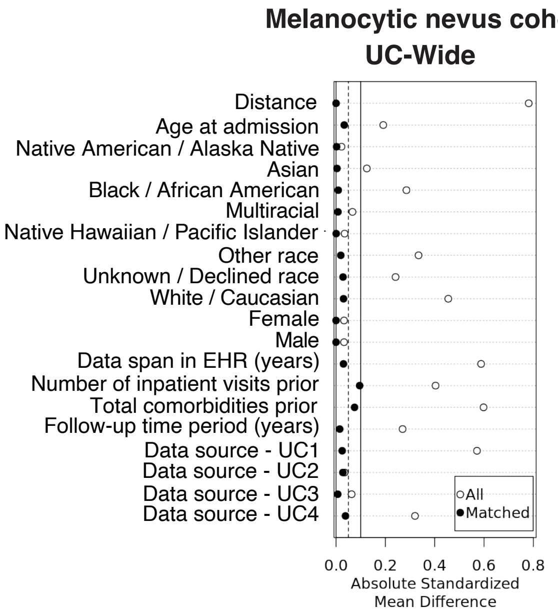

c.

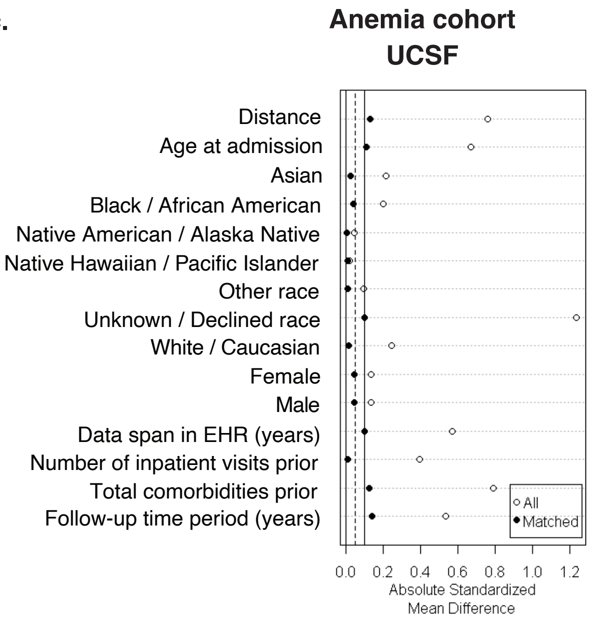

d.

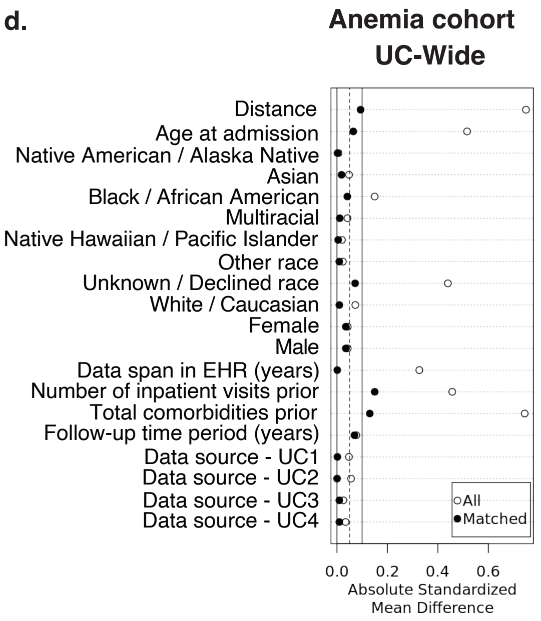

e.

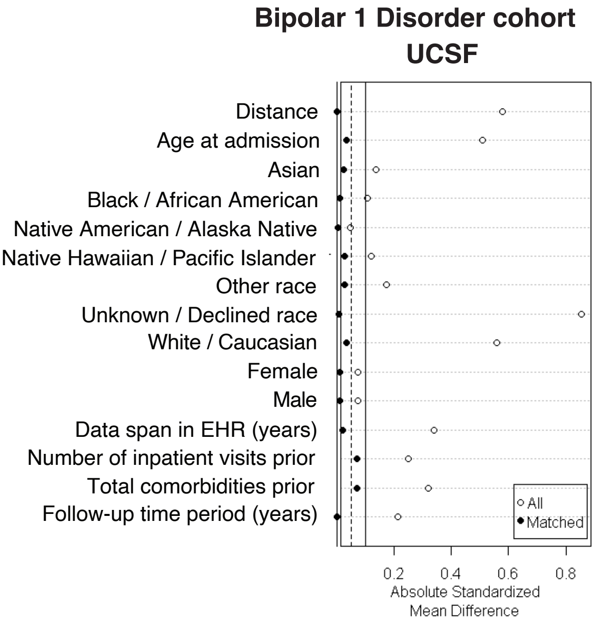

f.

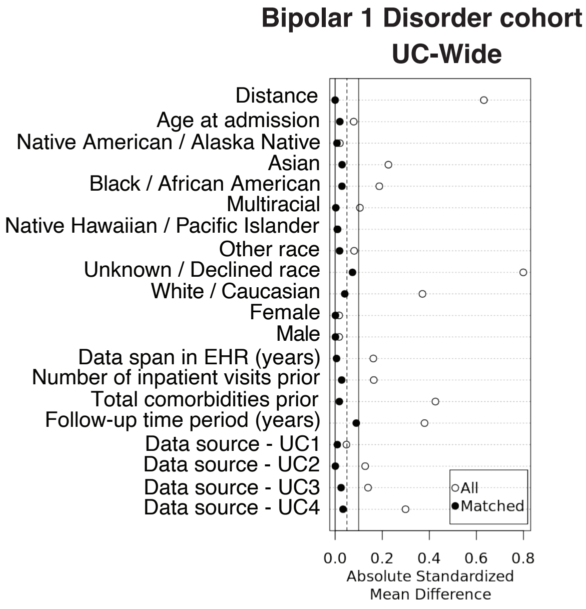

Supplementary Figure 7 (related to Fig 5). Time from diagnostic risk factor to delirium in UC-wide data

a.

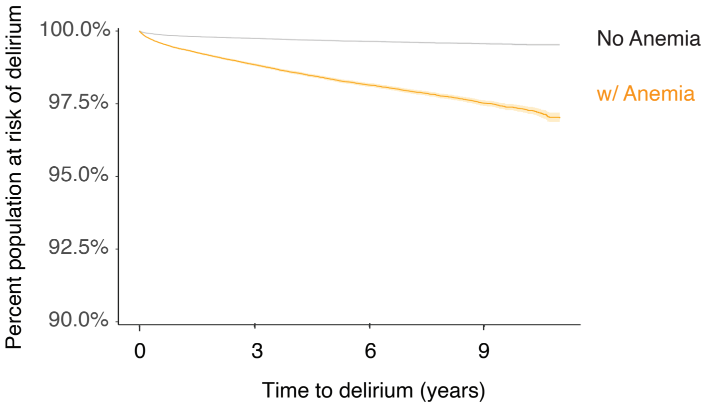

**At Risk**				
No Anemia	272023	163619	91826	30602
w/ Anemia	272651	154017	83211	29294

**Delirium**				
No Anemia	0	594	729	778
w/ Anemia	0	2533	3382	3735

b.

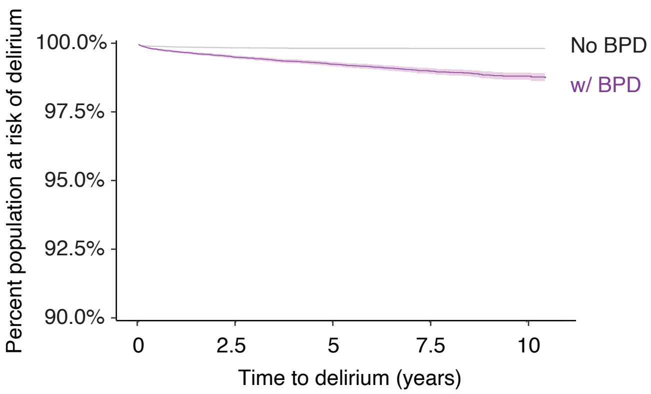

**At Risk**					
No BPD	57331	40283	29779	18642	6216
w/ BPD	57489	41162	27331	14418	3781

**Delirium**					
No BPD	0	67	73	74	74
w/ BPD	0	239	326	377	396

c.

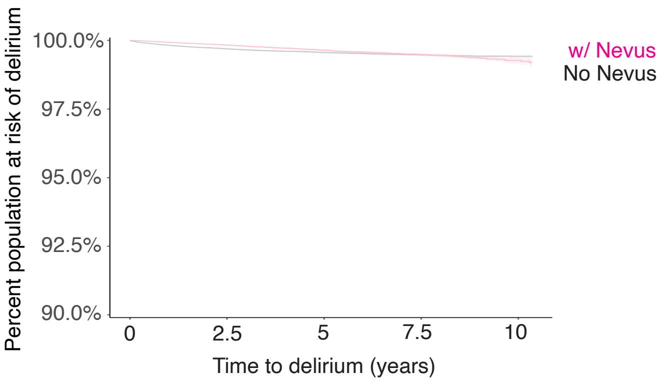

**At Risk**					
No Nevus	155399	106537	72060	39905	10022
w/ Nevus	155399	112170	72745	34133	3011

**Delirium**					
No Nevus	1	413	537	586	602
w/ Nevus	0	229	404	492	526

Kaplan-Meier curve showing time-to-event where event is defined as delirium, death, or loss to follow-up since the first-time diagnosis of anemia (a), bipolar disorder (b), and melanocytic nevus (c) in UC-wide patients.
