# Supplementary material for: Comorbidities associated with a clinically-recognized delirium diagnosis in the hospital using real world data
Source: Commun Med (Lond). 2025 Jul 22;5:304. doi: 10.1038/s43856-025-00986-5 (PMC12284072; doi:10.1038/s43856-025-00986-5)
Supplement: Supplementary file 2 — Description of Additional Supplementary Files [file 43856_2025_986_MOESM2_ESM.pdf]

Description of Additional Supplementary Files:

Supplementary Tables:

Supplementary Data 1: Table of demographic information for patient cohorts identified in UCSF (left) and UC-wide (right) data. Chi-squared test used for categorical measures. Student's t-test used for continuous measures. SMD = standardized mean difference. UC = University of California. UCSF = University of California San Francisco.

Supplementary Data 2 (Related to Fig. 2): Differential comorbidities between delirium and control patients in University of California San Francisco (UCSF) data

Supplementary Data 3 (Related to Fig. 2): Differential comorbidities between delirium and control patients in University of California (UC)-wide data

Supplementary Data 4 (Related to Fig. 2): Differential comorbidities between delirium and control patients significant in both datasets

Supplementary Data 5 (Related to Fig. 3): Differential laboratory median values in UCSF and UC-wide data

Supplementary Data 6 (Related to Fig. 4): Sex-stratified differential comorbidities between delirium and control patients significant in University of California San Francisco (UCSF) data

Supplementary Data 7 (Related to Fig. 4): Sex-stratified differential comorbidities between delirium and control patients significant in University of California (UC)-wide data
