# Supplementary material for: Comorbidities associated with a clinically-recognized delirium diagnosis in the hospital using real world data
Source: Commun Med (Lond). 2025 Jul 22;5:304. doi: 10.1038/s43856-025-00986-5 (PMC12284072; doi:10.1038/s43856-025-00986-5)
Supplement: Supplementary file 10 — Reporting Summary [file 43856_2025_986_MOESM10_ESM.pdf]

nature portfolio

Corresponding author(s): Sirota, Marina

Last updated by author(s): 05/13/2025

Reporting Summary

Nature Portfolio wishes to improve the reproducibility of the work that we publish. This form provides structure for consistency and transparency in reporting. For further information on Nature Portfolio policies, see our Editorial Policies and the Editorial Policy Checklist.

Statistics

For all statistical analyses, confirm that the following items are present in the figure legend, table legend, main text, or Methods section.

n/a	Confirmed	
☐	☒	The exact sample size (*n*) for each experimental group/condition, given as a discrete number and unit of measurement
☐	☒	A statement on whether measurements were taken from distinct samples or whether the same sample was measured repeatedly
☐	☒	The statistical test(s) used AND whether they are one- or two-sided<br>*Only common tests should be described solely by name; describe more complex techniques in the Methods section.*
☐	☒	A description of all covariates tested
☐	☒	A description of any assumptions or corrections, such as tests of normality and adjustment for multiple comparisons
☐	☒	A full description of the statistical parameters including central tendency (e.g. means) or other basic estimates (e.g. regression coefficient) AND variation (e.g. standard deviation) or associated estimates of uncertainty (e.g. confidence intervals)
☐	☒	For null hypothesis testing, the test statistic (e.g. *F*, *t*, *r*) with confidence intervals, effect sizes, degrees of freedom and *P* value noted<br>*Give P values as exact values whenever suitable.*
☒	☐	For Bayesian analysis, information on the choice of priors and Markov chain Monte Carlo settings
☒	☐	For hierarchical and complex designs, identification of the appropriate level for tests and full reporting of outcomes
☒	☐	Estimates of effect sizes (e.g. Cohen's *d*, Pearson's *r*), indicating how they were calculated

Our web collection on statistics for biologists contains articles on many of the points above.

Software and code

Policy information about availability of computer code

Data collection	No new data was collected for this study. The UCSF EHR database is available to individuals affiliated with UCSF who can contact the UCSF's Clinical and Translational Science Institute (CTSI) (ctsi@ucsf.edu) or the UCSF's Information Commons team for more information (Info.Commons@ucsf.edu). The UC-wide EHR database is only available to UC researchers who have completed analyses in their respective UC first and have provided justification for scaling their analyses across UC health centers (more details at https://www.ucop.edu/uc-health/departments/center-for-data-driven-insights-and-innovations-cdi2.html or by contacting healthdata@ucop.edu.
Data analysis	The code used for analysis is available on GitHub at https://github.com/kozlama/delirium_EHR. Any additional information required to reanalyze the data reported in this paper is available from the lead contact upon request.

For manuscripts utilizing custom algorithms or software that are central to the research but not yet described in published literature, software must be made available to editors and reviewers. We strongly encourage code deposition in a community repository (e.g. GitHub). See the Nature Portfolio guidelines for submitting code & software for further information.

Data

Policy information about availability of data

All manuscripts must include a data availability statement. This statement should provide the following information, where applicable:

- Accession codes, unique identifiers, or web links for publicly available datasets
- A description of any restrictions on data availability
- For clinical datasets or third party data, please ensure that the statement adheres to our policy

The UCSF EHR database is available to individuals affiliated with UCSF who can contact the UCSF's Clinical and Translational Science Institute (CTSI) (ctsi@ucsf.edu) or the UCSF's Information Commons team for more information (Info.Commons@ucsf.edu). The UC-wide EHR database is only available to UC researchers who have completed analyses in their respective UC first and have provided justification for scaling their analyses across UC health centers (more details at https://www.ucop.edu/uc-health/departments/center-for-data-driven-insights-and-innovations-cdi2.html or by contacting healthdata@ucop.edu.

Research involving human participants, their data, or biological material

Policy information about studies with human participants or human data. See also policy information about sex, gender (identity/presentation), and sexual orientation and race, ethnicity and racism.

Reporting on sex and gender	In this study, the definition of sex in the EHR is likely a combination of sex assigned at birth, legal sex, and sex determined by the clinician. Documentation of gender identity remains a challenge in the EHR and only recently has garnered attention to provide more inclusive and affirmative health care for all patients.
Reporting on race, ethnicity, or other socially relevant groupings	In this study, the definition of race was used through self-reported, self-identified race as documented in the EHR.
Population characteristics	Population characteristics such as demographic information and their summaries can be found in Table 1 of the manuscript.
Recruitment	No recruitment process. Data was pulled from the electronic health record from UC health centers. All clinical data were de-identified and written informed consent was waived by the institutions. Patient cohorts were identified using the UCSF de-identified and UC-wide HIPAA-compliant limited data set OMOP EHR databases. The UCSF dataset included over five million patients from January 1, 1982 to February 20, 2023 while the UC-wide dataset included over seven million patients from January 1, 2012 to April 19, 2023 from 4 sites (UC San Diego, UC Los Angeles, UC Irvine, and UC Davis).
Ethics oversight	All analysis of UCSF and UC-wide EHR data was performed under the approval of the Institutional Review Boards.

Note that full information on the approval of the study protocol must also be provided in the manuscript.

Field-specific reporting

Please select the one below that is the best fit for your research. If you are not sure, read the appropriate sections before making your selection.

☐ Life sciences ☒ Behavioural & social sciences ☐ Ecological, evolutionary & environmental sciences

For a reference copy of the document with all sections, see nature.com/documents/nr-reporting-summary-flat.pdf

Behavioural & social sciences study design

All studies must disclose on these points even when the disclosure is negative.

Study description	This study utilized data from the electronic health record and is an observational study.
Research sample	All clinical data were de-identified and written informed consent was waived by the institutions. Patient cohorts were identified using the UCSF de-identified and UC-wide HIPAA-compliant limited data set OMOP EHR databases. The UCSF dataset included over five million patients from January 1, 1982 to February 20, 2023 while the UC-wide dataset included over seven million patients from January 1, 2012 to April 19, 2023 from 4 sites (UC San Diego, UC Los Angeles, UC Irvine, and UC Davis). Patients with inpatient delirium were identified using the OMOP concept ID 373995, SNOMED code 2776000 (corresponding to "Delirium"), filtered for first-time diagnosis of delirium during an inpatient stay (i.e., 'visit of interest'). Control patients were matched 1:1 using propensity score matching as described in the methods. Demographic information about the study population is summarized in Table 1.
Sampling strategy	Patient with delirium were identified using the diagnostic filter above. Control patients were identified from the rest of the remaining pool of patients in the dataset, using propensity score matching at 1:1 ratio using a nearest neighbor method and the following matching criteria: assigned sex, patient-reported race, estimated age at admission, years in EHR prior to visit, total number of comorbidities and inpatient visits prior to the visit of interest, stay length, stay type (ICU vs non-ICU), death during admission, and UC location (for UC-wide dataset).
Data collection	No new data were collected for this study.

Timing	No new data were collected for this study.
Data exclusions	For the analysis of differential laboratory test results, tests that had more than 95% of patients missing the lab test results were excluded from the analysis. Patients with no value available for a lab test of interest were also excluded from the analysis. These parameters were adapted from a prior study (Tang et al. 2022).
Non-participation	No new data were collected for this study.
Randomization	This study did not randomize patients into groups.

Reporting for specific materials, systems and methods

We require information from authors about some types of materials, experimental systems and methods used in many studies. Here, indicate whether each material, system or method listed is relevant to your study. If you are not sure if a list item applies to your research, read the appropriate section before selecting a response.

Materials & experimental systems

n/a	Involved in the study
☒	☐ Antibodies
☒	☐ Eukaryotic cell lines
☒	☐ Palaeontology and archaeology
☒	☐ Animals and other organisms
☒	☐ Clinical data
☒	☐ Dual use research of concern
☒	☐ Plants

Methods

n/a	Involved in the study
☒	☐ ChIP-seq
☒	☐ Flow cytometry
☒	☐ MRI-based neuroimaging

Plants

Seed stocks	*Report on the source of all seed stocks or other plant material used. If applicable, state the seed stock centre and catalogue number. If plant specimens were collected from the field, describe the collection location, date and sampling procedures.*
Novel plant genotypes	*Describe the methods by which all novel plant genotypes were produced. This includes those generated by transgenic approaches, gene editing, chemical/radiation-based mutagenesis and hybridization. For transgenic lines, describe the transformation method, the number of independent lines analyzed and the generation upon which experiments were performed. For gene-edited lines, describe the editor used, the endogenous sequence targeted for editing, the targeting guide RNA sequence (if applicable) and how the editor was applied.*
Authentication	*Describe any authentication procedures for each seed stock used or novel genotype generated. Describe any experiments used to assess the effect of a mutation and, where applicable, how potential secondary effects (e.g. second site T-DNA insertions, mosiacism, off-target gene editing) were examined.*
